# Molecular Modelling and Simulations of Light‐Harvesting Decanuclear Ru‐Based Dendrimers for Artificial Photosynthesis

**DOI:** 10.1002/chem.202103310

**Published:** 2021-12-16

**Authors:** Giovanna M. A. Rogati, Chiara Capecci, Enza Fazio, Scolastica Serroni, Fausto Puntoriero, Sebastiano Campagna, Leonardo Guidoni

**Affiliations:** ^1^ Dipartimento di Ingegneria Scienze dell'Informazione e Matematica Università dell'Aquila Via Vetoio 2, Coppito 67100 L'Aquila Italy; ^2^ Dipartimento di Fisica Università di Roma La Sapienza Piazzale Aldo Moro, 5 00185 Roma Italy; ^3^ Dipartimento di Scienze Matematiche e Informatiche Scienze Fisiche e Scienze della Terra Università di Messina Piazza Pugliatti, 1 98122 Messina Italy; ^4^ Dipartimento di Scienze Chimiche, Biologiche, Farmaceutiche ed Ambientali Università di Messina Piazza Pugliatti, 1 98122 Messina Italy; ^5^ Dipartimento di Scienze Fisiche e Chimiche Università dell'Aquila Via Vetoio, 2, Coppito 67100 L'Aquila Italy

**Keywords:** computational chemistry, dendrimers, molecular simulations, ruthenium

## Abstract

The structure of a decanuclear photo‐ and redox‐active dendrimer based on Ru(II) polypyridine subunits, suitable as a light‐harvesting multicomponent species for artificial photosynthesis, has been investigated by means of computer modelling. The compound has the general formula [Ru{(μ‐dpp)Ru[(μ‐dpp)Ru(bpy)_2_]_2_}_3_](PF_6_)_20_ (**Ru10**; bpy=2,2′‐bipyridine; dpp=2,3‐bis(2′‐pyridyl)pyrazine). The stability of possible isomers of each monomer was investigated by performing classical molecular dynamics (MD) and quantum mechanics (QM) simulations on each monomer and comparing the results. The number of stable isomers is reduced to 36 with a prevalence of MER isomerism in the central core, as previously observed by NMR experiments. The simulations on decanuclear dendrimers suggest that the stability of the dendrimer is not linked to the stability of the individual monomers composing the dendrimer but rather governed by the steric constrains originated by the multimetallic assembly. Finally, the self‐aggregation of **Ru10** and the distribution of the counterions around the complexes is investigated using Molecular Dynamics both in implicit and explicit acetonitrile solution. In representative examples, with nine and four dendrimers, the calculated pair distribution function for the ruthenium centers suggests a self‐aggregation mechanism in which the dendrimers are approaching in small blocks and then aggregate all together. Scanning transmission electron microscopy complements the investigation, supporting the formation of different aggregates at various concentrations.

## Graphical abstract

**Molucular simulations** on the structure of a decanuclear Ru‐based dendrimer capable to play the role of antenna for artificial photosynthesis is performed. Aggregation of dendrimers is also modelled and compared to experimental results obtained by SAXS, scanning tunneling electron microscopy and energy‐dispersive X‐ray analysis.

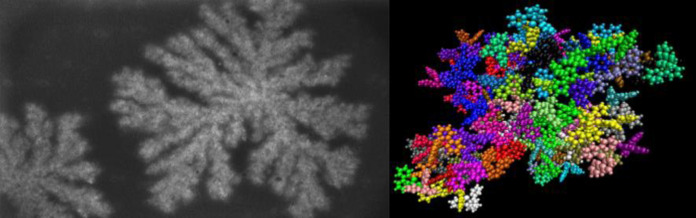

## Introduction

Artificial photosynthesis aims to design multicomponent supramolecular systems for performing conversion of raw species of low‐energy content, such as CO_2_ and water, into high‐energy chemicals, such as molecular oxygen and hydrogen or reduced forms of CO_2_, by using solar energy as the energy source, operating at the molecular level.^[1]^ Motivation for this research has its roots in the increasing demand for sustainable energy sources, as a direct consequence of the growing needs for energy at the global level – expected to double by 2050 – and the evident effects of global warming, alimented by the use of fossil fuel energy sources, that severely impact environmental and societal issues.^[2]^

**Scheme 1:**
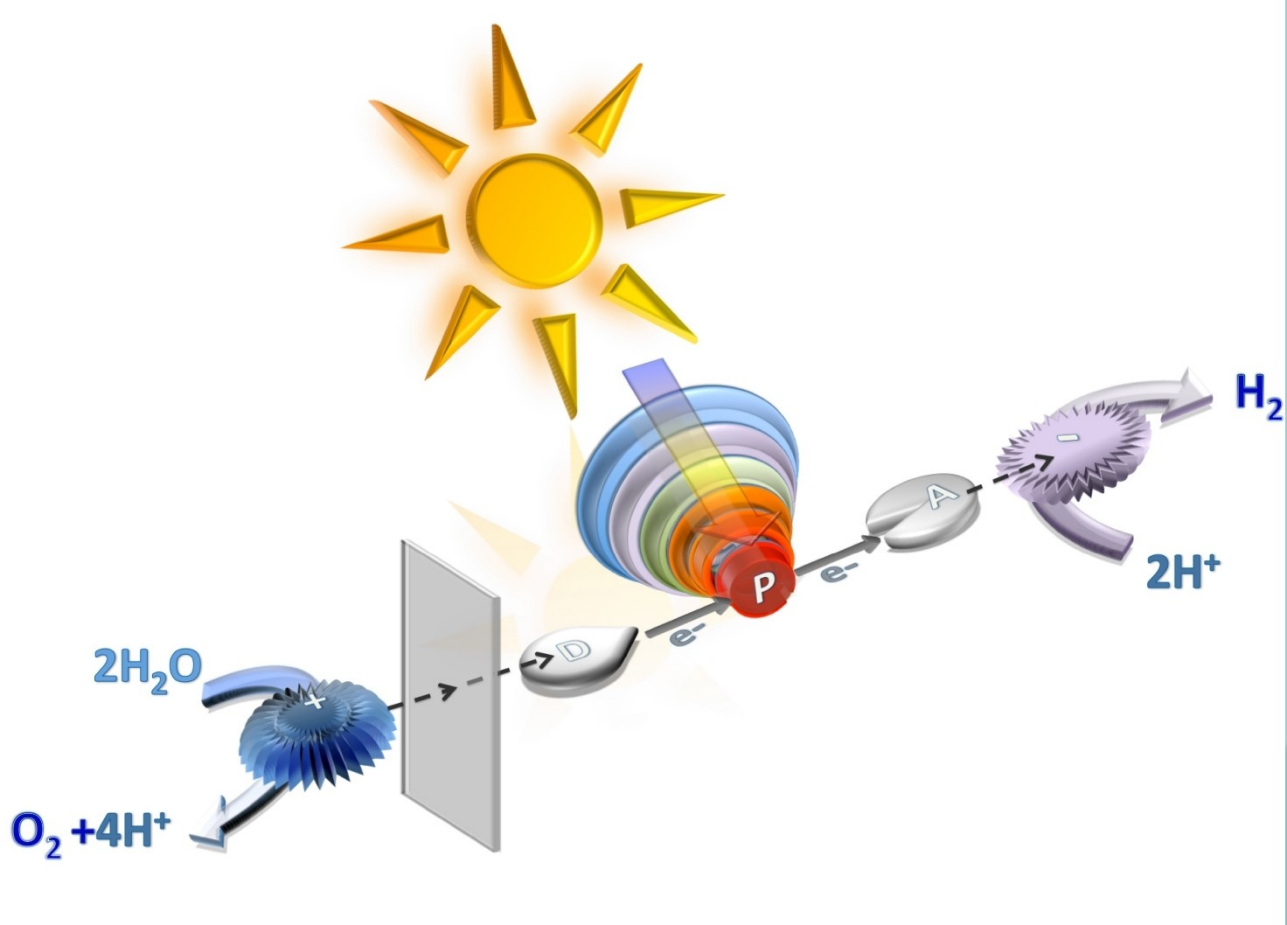
A representation of an artificial photosynthesis system for photoinduced water splitting.

In analogy with the natural systems, artificial photosynthetic systems have to (i) collect light energy (the role is performed by the so‐called antenna systems), (ii) separate charges (role played by the so‐called reaction centers), (iii) transport and accumulate electrons or holes in catalytic sites capable to drive multielectron redox processes (e. g., water splitting, with formation of molecular oxygen on the oxidative side and molecular hydrogen on the reductive side) (see Scheme 1). From the synthetic viewpoint, very interesting assemblies of specific subunits have been prepared by using covalent linkages,^[3]^ as well as different subunits have been assembled on electrodes and surfaces in a more or less controlled way.^[4]^ However, all these above‐mentioned synthetic approaches, although quite useful to obtain systems suitable to investigate mechanistic details and potentially useful for applications, are still less effective than the self‐assembly strategies developed by Nature. In recent years, progress towards the development of self‐assembled photofunctional structures in solution has been obtained by integrating light‐harvesting and charge separation subunits using strategies mainly based on π‐stacking[^5^, ^6^] and/or metal‐ligand interaction.^[7]^ Large efforts for designing self‐assembled light‐harvesting antenna systems have been made, however in most cases the results involve assemblies of single chromophores into multicomponent arrays. Examples of pre‐formed multichromophoric systems joined by successive self‐assembly are extremely rare, and – with the exception of seminal works on hydrogen‐bonded^[8]^ and specific host‐guest species,^[9]^ mainly involve the use of additional components, like polymers, to generate scaffolds in which the multichromoporic species are physically constrained.[^10^, ^11^, ^12^]

In the last decades we have developed covalently‐linked photo‐ and redox‐active metal dendrimers made of Ru(II) and Os(II) polypyridine building blocks and investigated the energy and electron transfer occurring within such multichromophoric systems.^[13]^ A first‐generation dendrimer of this class of compounds has also been profitably employed as antenna systems for photoinduced water oxidation purposes, leading to outstanding quantum yields for this process and allowing to make in evidence some peculiarities of the photoinduced mechanism.^[14]^ Spontaneous assemblies of such dendrimers with metal catalysts for water oxidation has also been reported.^[15]^ Very recently, we have demonstrated that second‐generation, decanuclear metal dendrimers of this class of compounds, with general formulas [Ru{(μ‐dpp)Ru[(μ‐dpp)Ru(bpy)_2_]_2_}_3_](PF_6_)_20_ (**Ru10**) and [Os{(μ‐dpp)Ru[(μ‐dpp)Ru(bpy)_2_]_2_}_3_](PF_6_)_20_ (**OsRu9**) (bpy=2,2′‐bipyridine; dpp=2,3‐bis(2′‐pyridyl)pyrazine), spontaneously aggregate in solution at relatively mild concentrations to form someway organized superstructures, in which new energy transfer pathways are also activated at a critical concentration.^[16]^ Such aggregates of light‐harvesting antenna dendrimers functionally resemble the behavior exhibited by LH1 and LH2 systems of photosynthetic bacteria, in which intra‐assembly and inter‐assembly energy transfer can take place.[^3e^, ^16^, ^17^]

Here we report a computational study aimed to rationalize the isomer composition and the spontaneous aggregation of **Ru10** dendrimer, starting from a basic discussion on the metal‐coordination steps involved in the construction of the multi‐ruthenium dendrimers, containing an unprecedented (to the best of our knowledge) analysis of possible isomeric composition, and leading to the self‐aggregation processes of the preformed dendrimers into larger aggregates. Although multi‐ruthenium(II) polypyridine complexes have been extensively investigated in the last decades, this is the first time (to the best of our knowledge) that a computational analysis is applied to characterize species containing more than four Ru(II) polypyridine centers. A scanning transmission electron microscopy (STEM) study complements the investigation, supporting the formation of different aggregates at various concentrations, as indicated by the photophysical experiments formerly reported.^[16]^

## Dendrimer Structures. Basic Considerations

**Figure 1:**
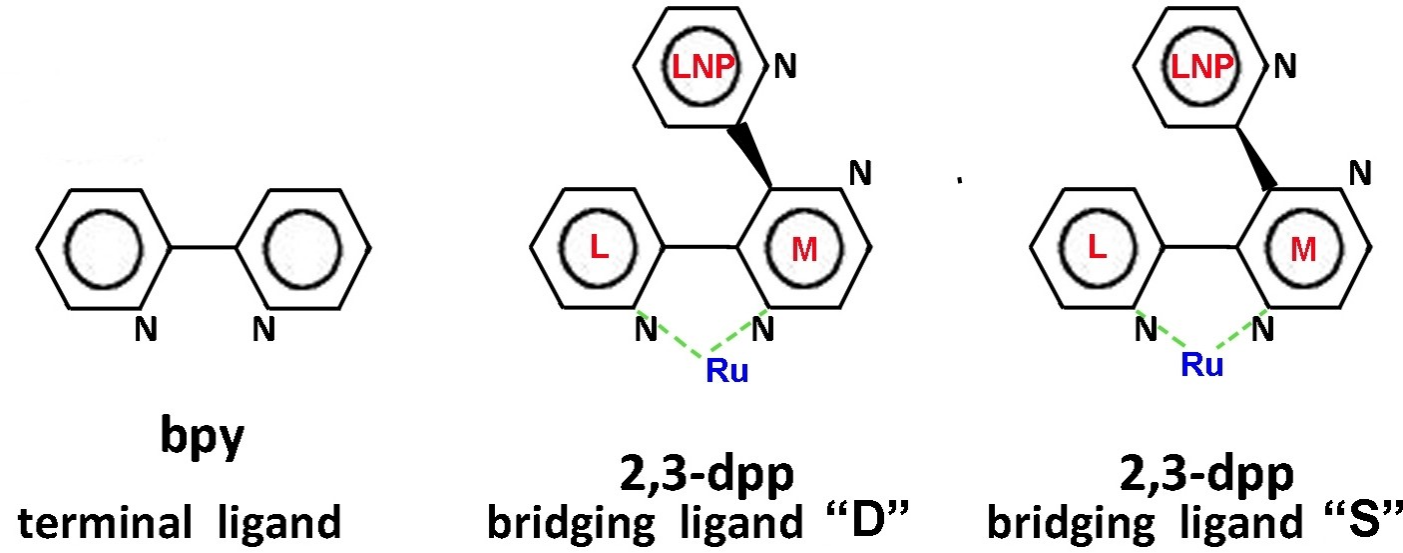
From left to right: a terminal ligand which contains one free chelating site (one free nitrogen pair) and the ′′D′′ and ′′S′′ bridging ligands that contains two free chelating sites. The two tridimensional spatial structures ′′D′′ and ′′S′′ of the dpp bridging ligand are due to repulsion between two hydrogen atoms of neighboring side rings. M indicates the central ring (*Medium)*, L indicates the lateral ring (*Lateral)* lying on the same plane of M, finally LNP indicates the lateral ring that does not lie on the same plane of the other two (*Lateral Non Planar*). We have called these structures D and S by analogy, respectively, with the right and the left hand (in latin *Dextra* and *Sinistra*): the LNP non‐planar ring represents the thumb, the M and L remaining rings represent, respectively, the palm and the other fingers of the hand. When the LNP non‐planar ring is above the plane containing the other two, we obtain the D structure (resembling a right hand with the palm upwards), otherwise, we obtain the S structure (resembling a left hand with the palm downwards). Whereas D and S are conformers for mononuclear and dinuclear compounds, in larger systems they can give arise to quite different structures.

The process of identifying possible isomers is the first hard challenge faced in metal dendrimers characterization. Metal dendrimers such as the **Ru10** species here investigated are highly branched tree‐like species formed by one metal atom (in our case, ruthenium) connected by bridging ligands to other metal nuclei (in our case, other ruthenium centers). Such metal dendrimers can be prepared by iterative protection/deprotection synthetic protocols, and the iterative grow process ends when terminal ligands are inserted.[^13c^, ^13d^, ^13e^] In our case, the bridging ligands are 2,3‐bis(2‐pyridyl)pyrazine (dpp), which contains two free chelating sites, while the terminal ligands are 2,2′‐bipyridine (bpy), which contains only one free chelating site. Figure 1 shows the chemical structure of the ligands. Due to the octahedral nature of the metal centers and the structure of dpp ligands, a very large number of geometrical and optical isomers are possible in a decanuclear compound. As detailed hereafter, we have studied a selection of such geometrical isomers and conformers, chosen on the basis of the modelling results. We do not take into consideration the optical Δ and Λ stereoisomers of each monomer, and implicitly select a single stereoisomer for each building block.

**Figure 2:**
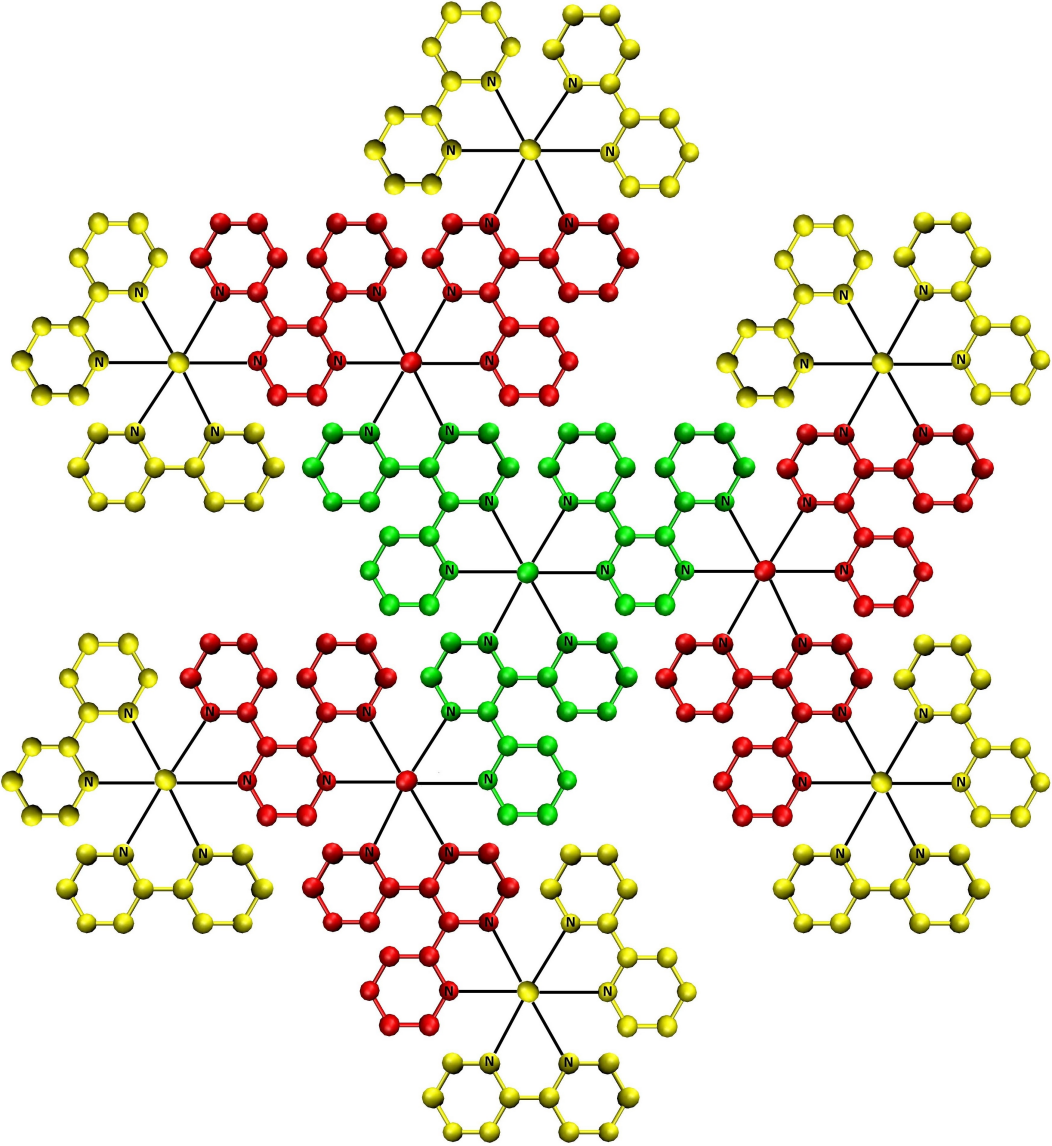
The structure of a decanuclear dendrimer (**Ru10**): one central monomer, formed by one Ru atom and three dpp ligands (green), three intermediate monomers, formed by one Ru atom and two dpp ligands (red), and six peripheral centers, formed by one Ru atom and two bpy ligands (yellow).

The decanuclear dendrimer **Ru10** consists of 10 monomers, as schematized in Figure 2:

one central core, represented in green (formed by one Ru+3 dpp ligands),
three intermediate cores, represented in red (formed by one Ru+2 dpp ligands),
six peripheral cores, represented in yellow (formed by one Ru+2 bpy ligands).

Each monomer/building block can indeed exist as different geometrical isomers depending on:

the arrangement of the ligands around the metal ions,
the spatial structure of the bridging ligands.

**Figure 3:**
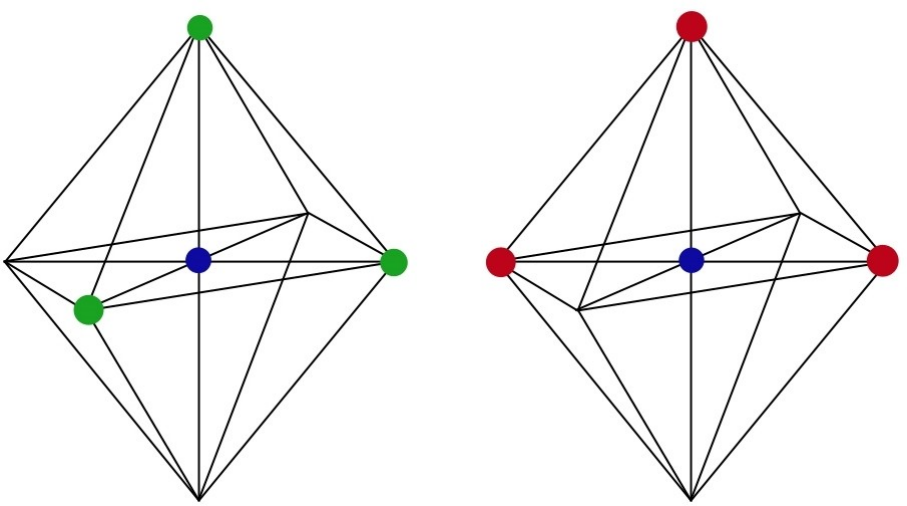
Facial (FAC, left) and meridional (MER, right) isomers of an octahedral metal complex.

The Ru(II) core binds the ligands according to an octahedral structure. When the pyrazine rings of the three chelating dpp ligands occupy the vertices of one face of the octahedron, facial isomers (FAC) are formed; if instead the pyrazine rings of the three dpp bridging ligands and the metal ion are on one plane, meridional isomers (MER) are formed, see Figure 3.

In addition, the bridging ligand dpp can exist as two different tridimensional structures (non‐planar) due to repulsion between two hydrogen atoms of neighboring side rings: one dpp side ring (LNP in Figure 1) is “pushed” above or below the other two (Figure 1). We have called these structures D and S by analogy, respectively, with the right and the left hand (in latin *Dextra* and *Sinistra*): the LNP non‐planar ring represents the thumb, the M (pyrazine) and L (pyridine) remaining rings represent, respectively, the palm and the other fingers of the hand. When the LNP non‐planar ring is above the plane containing the other two, we obtain the D structure (resembling a right hand with the palm upwards), instead if the LNP non‐planar ring is under the plane containing the other two, we obtain the S structure (resembling a left hand with the palm downwards). Please note that D and S forms are conformers, however they can have a strong impact on the general shape of the dendrimers. Moreover, whereas the rotation ables to convert one conformer into the other is relatively low‐energy demanding in the case of mononuclear compounds, it can become high‐energy demanding in polynuclear complexes, where dpp bridges two (multi)nuclear species, therefore we consider them as real different isomers.

**Figure 4:**
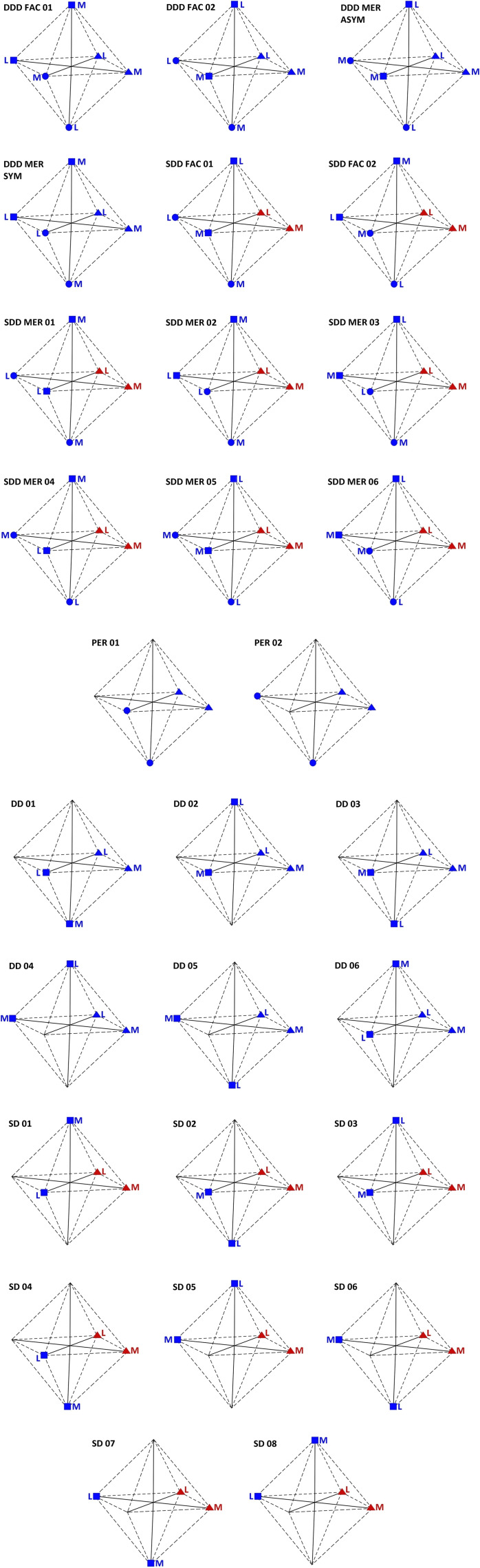
In the boxes from top to bottom, respectively: the schemes of 12 central, 2 peripheral and 14 intermediate blocks (the enantiomers are not shown). M and L represent respectively the central and the side ring of dpp (Figure 1). The red symbols indicate the S‐dpp, instead the blue‐ones indicate the D‐dpp (Figure 1). Equal (circles and triangles) symbols represent the attack points of the same dpp, equal yellow symbols represent the attack points of the same bpy terminal ligand.

Overall we have identified (see Figure 4):

12 isomers of the central core (plus 12 related enantiomers, not shown in Figure 4): four configurations DDD (two of which have isomerism FAC) with three D‐dpp and eight configurations SDD (two of which have isomerism FAC) with two D‐dpp and one S‐dpp ligands;
14 isomers of the intermediate core (plus 6 related enantiomers, not shown in Figure 4): six configurations DD with two D‐dpp and eight configurations SD with one D‐dpp and one S‐dpp ligands;
2 isomers of peripheral cores (the terminal ligands are “planar”).

## Methods Employed

We have performed molecular dynamics (MD) and quantum mechanics (QM) simulations on every monomer structure to investigate their stability and to consequently reduce the number of isomers from which we may choose the components of the dendrimer.

### MD Simulations

All the MD simulation were carried out using AMBER 14 package^[18]^ using SANDER program. We used the AMBER/parm99 force field, supplemented by the parameters developed by Norrby et al.^[19]^ for ruthenium(II) polypyridyl compounds, to which appropriate unit corrections were applied.^[20]^ Starting coordinates and partial charges were obtained from DFT‐optimised geometries.

In preliminary MD‐optimized structures of monomers, it can be noted that some pyrazine rings (M in Figure 1) of the dpp were distorted (some rings appear non‐planar, therefore deformed, as also reported in Figure 1 of Supporting Information). This distortion is due to the chosen parameters of the force field, in particular because of those related to the dihedral angle N−C−C−N. For this reason, the force field related to this dihedral angle N−C−C−N has been reparameterized. In order to obtain the torsion angle parameters, we have fitted the data obtained by subtracting the results of the QM simulations with relaxed surface scan (DFT calculations with partial charges obtained with Conductor‐like Polarizable Continuum Model with COSMO epsilon function for implicit solvent: acetonitrile dielectric constant *ϵ*=36.6. Further details on reparameterization and applied parameter values can be found in Supporting Information) to those of the MD simulations with scan on dihedral angle we were interested in, but with C−C−C−C dihedral force constant reset to zero.[^21^, ^22^] The additional parameters used in the simulation are reported in the Table 1 of Supporting Information.

### Molecular dynamics simulations of dendrimers in explicit solvent

We inserted the dendrimer geometry optimized in gas phase in a cubic box with edge l=60 Å. In order to neutralize the dendrimer charge +20 due to ten Ru(II) ions, we added twenty hexafluorophosphate anions, PF_6_^−^, using packmol package.^[23]^ The solutions were composed by 2088 molecules of acetonitrile. An equilibration run of 50 ns was carried out at room temperature.

### Molecular dynamics simulations of dendrimer groups

To investigate the process of self‐assembling of different dendrimers we have selected two different modelling scenarios in a cubic box with edge l=100 Å:

a system with 9 dendrimers, all different from each other (randomly chosen from the 10 we had built, described below), initially arranged close to the center and vertices of the box, with 180 PF_6_^−^ counterions, was simulated using implicit solvent (acetonitrile, ϵ=36.6) in NVT ensemble for a total time of 200 ns at a temperature of 300 K.
a system with 4 dendrimers, all the same type (SDD MER 05+SD02+DD03+SD06) in a box with explicit acetonitrile solvent (7032 acetonitrile molecules) initially located at the maximum allowed distance, with 80 PF_6_^−^ counterions, was simulated in NPT ensemble for a total time of 50 ns at a pressure of 1 atm and temperature of 300 K.

We also analyzed another group of 9 dendrimers composed by replicas of the dendrimer with the lowest energy: these results (both the movie of trajectories and the calculated pair distance distribution function) are not reported as they are absolutely similar to the reported case.

### QM Simulations

The QM simulations were performed using ORCA 4.1 package.^[24]^ The simulations and the data analysis are performed on HPC‐CALIBAN cluster (University of L'Aquila), CINECA HPC‐MARCONI and GALILEO. QM simulations were carried out first in order to use the optimized structures obtained as a starting configuration for the classical MD ones. We have performed DFT calculations with the following settings: B3LYP hybrid functional,[^25^, ^26^] RIJCOSX approximation algorithm,^[27]^ D3 method of dispersion correction.^[28]^ Basis set for N and Ru was def2‐TZVP, for C and H was def2‐SVP; integration grid 5; TIGHT‐SCF convergence criterium to obtain an accurate single point energy (energy change 1.0×10^−8^ au); slowconv (SCF converger criterium for difficult cases); CPCMC model (Conductor‐like Polarizable Continuum Model with COSMO epsilon function) for implicit solvent with acetonitrile dielectric constant: *ϵ*=36.6; multiplicity=2S+1=1. We have verified that the lowest energy is reached by singlet multiplicity.

### Dendrimer Models

The 10 “monomers” (i. e., the building blocks) that constitute each dendrimer were initially arranged according to the structure in which the central and the intermediate ruthenium atoms occupied the vertices of a triangular pyramid whereas the peripheral ruthenium atoms occupied the vertices of a triangular prism. To build the models of the full dendrimers we defined a MD protocol to guarantee that the final geometry of the polymer is compatible with the steric hyndrances imposed by the monomer‐monomer interaction. Hereafter is described such protocol.

Initially the ruthenium(II) ions of each subunit were positioned at a distance of about 16 Å from the other Ru ions to avoid overlapping. Then, we have generated a new topology file imposing a suitably weakened FF (force constant of N−Ru bond has been reduced by a factor of 10) and later we performed MD simulations:

we fixed the position of the Ru centers allowing to rotate ligands around the metal to reach a more suitable position of the free chelating site, NN, which should bind to the previous (or subsequent) ruthenium atom;
we get the monomers close to each other up to a N−Ru distance of 2.07 Å using a distance restraints between free N and Ru;
we performed a short equilibration run (20 ps) to reach the temperature of 300 K.

Finally, we created the bond between the free pairs NN and the Ru atoms in a new topology file (with the standard force constant of N−Ru bond in FF) in order to obtain the decanuclear dendrimer structure(s).

Observing the MD and QM optimized structures of monomers, we noticed that the nitrogen atoms of the free chelating site in some monomers were arranged at the opposite side (see an example at Figure 6 of the Supporting Information). We have therefore excluded these conformers because this condition prevents the creation of an additional bond with ruthenium to form a multinuclear (dendrimer) molecule. Henceforth the following structures will no longer be considered: DDD FAC 01, SDD MER 03, SDD MER 06, DD 04, DD 05, DD 07 and DD 08.

The “composed” decanuclear dendrimers were as follows (the peripheral subunits are not indicated):

**model 1**: DDD FAC 02+3DD05 (composed of central and intermediate monomers with the lowest energy)
**model 2**: DDD FAC 02+3SD06
**model 3**: DDD MER ASYM+3DD02
**model 4**: DDD MER ASYM+3SD02
**model 5**: DDD MER SYM+3DD06
**model 6**: SDD MER 01+3SD06
**model 7**: SDD MER 04+3DD06 (composed of central and intermediate monomers with the highest energy)
**model 8**: SDD FAC 01+3DD02
**model 9**: SDD MER 05+SD02+DD03+SD06
**model 10**: SDD MER 01+DD02+SD06+DD05

Each monomer model underwent a simulated annealing step of 380 ps, in which the temperature has been increased to 1000 K and then reduced to 300 K. The geometries of the post annealing structures were optimized in gas phase with an implicit solvent model.

## Results and Discussion

### Monomers/building blocks

**Figure 5:**
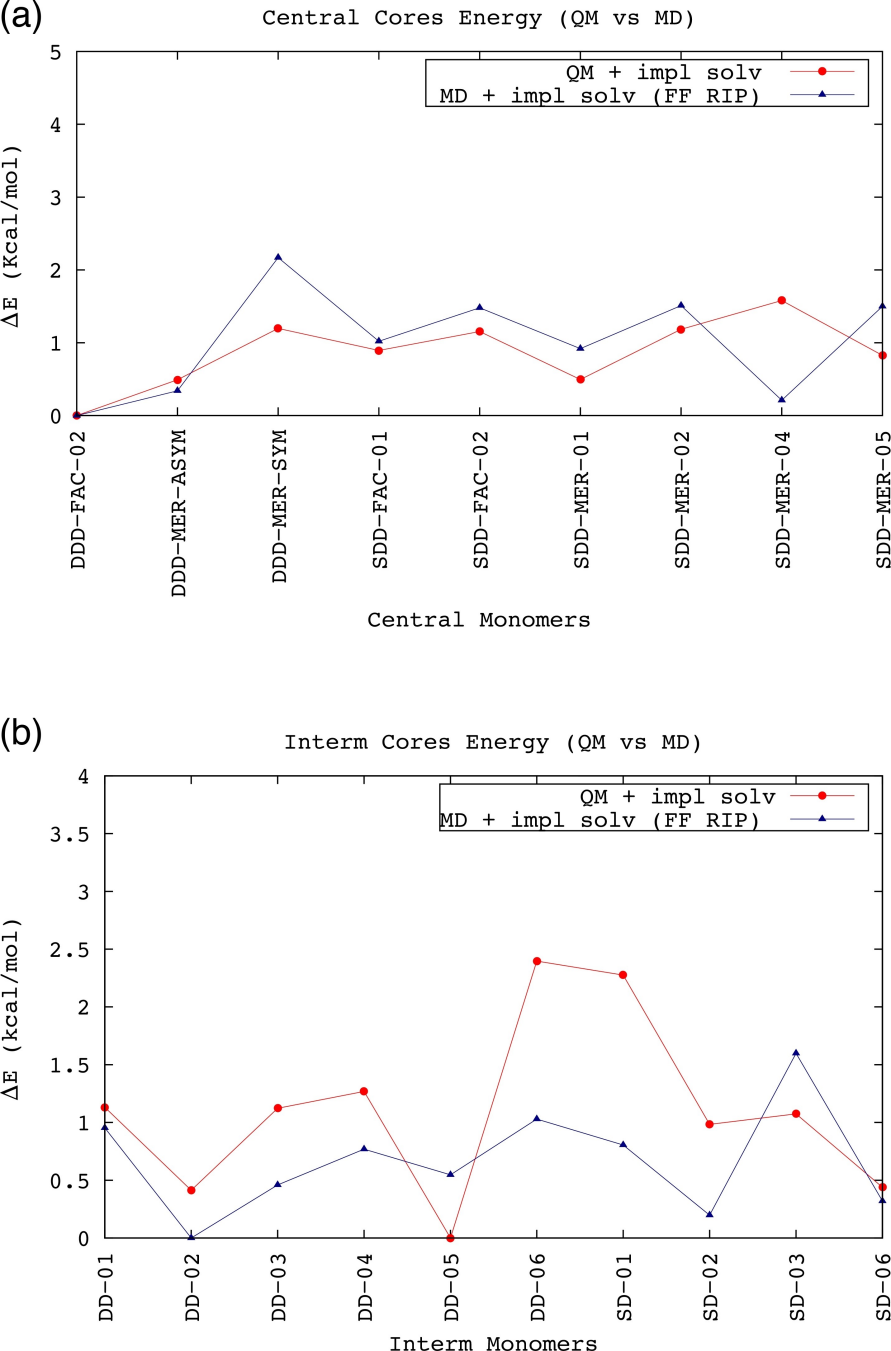
Comparison between the Δ*E* values (in kcal/mol) of central (a) and intermediate (b) isomers. the Δ*E* values were obtained by subtracting the value of isomer with the lowest energy from each monomer energy, respectively in QM simulations (red line) and MD ripametrized simulations (navy line). The prevalence of MER isomerism between the central blocks can be observed.

The energy differences between central and intermediate monomers was obtained using MD and QM simulations and reported in Figure 5. Both FAC and MER isomerism and the D and S conformations have been analysed. Unfortunately, the small energy differences among the isomers (less than 3 kcal/mol) did not allow us to exclude any of them.

Comparison between the results of QM and MD simulations provides a good match both for the central and intermediate monomers. The prevalence of MER isomerism, experimentally observed for the core building block,[^29^, ^30^] is confirmed. The match between the Δ*E* values of the intermediate cores according to QM and MD simulations is a bit worse, but the trend is similar (Figure 5b).

Finally, the comparison between the ΔE values of the peripheral cores PER01 and PER02, yields Δ*E* of 0.773 and 0.983 kcal mol^−1^ from QM and MD simulations, respectively. Such small values (less than 1 kcal mol^−1^ in both cases, with PER01 being the lowest‐energy isomer) did not allowed us to exclude one of the two peripheral isomers. The above results are anyway suggesting that the energetics of the single monomer is fairly described by the classical model itself, that will be therefore used to describe the conformers of the full dendrimers.

### Multimetallic Dendrimers

To investigate the structure and the energetics of decanuclear dendrimers we built 10 models combining different building blocks, following the procedure described in the Methods section.

**Figure 6:**
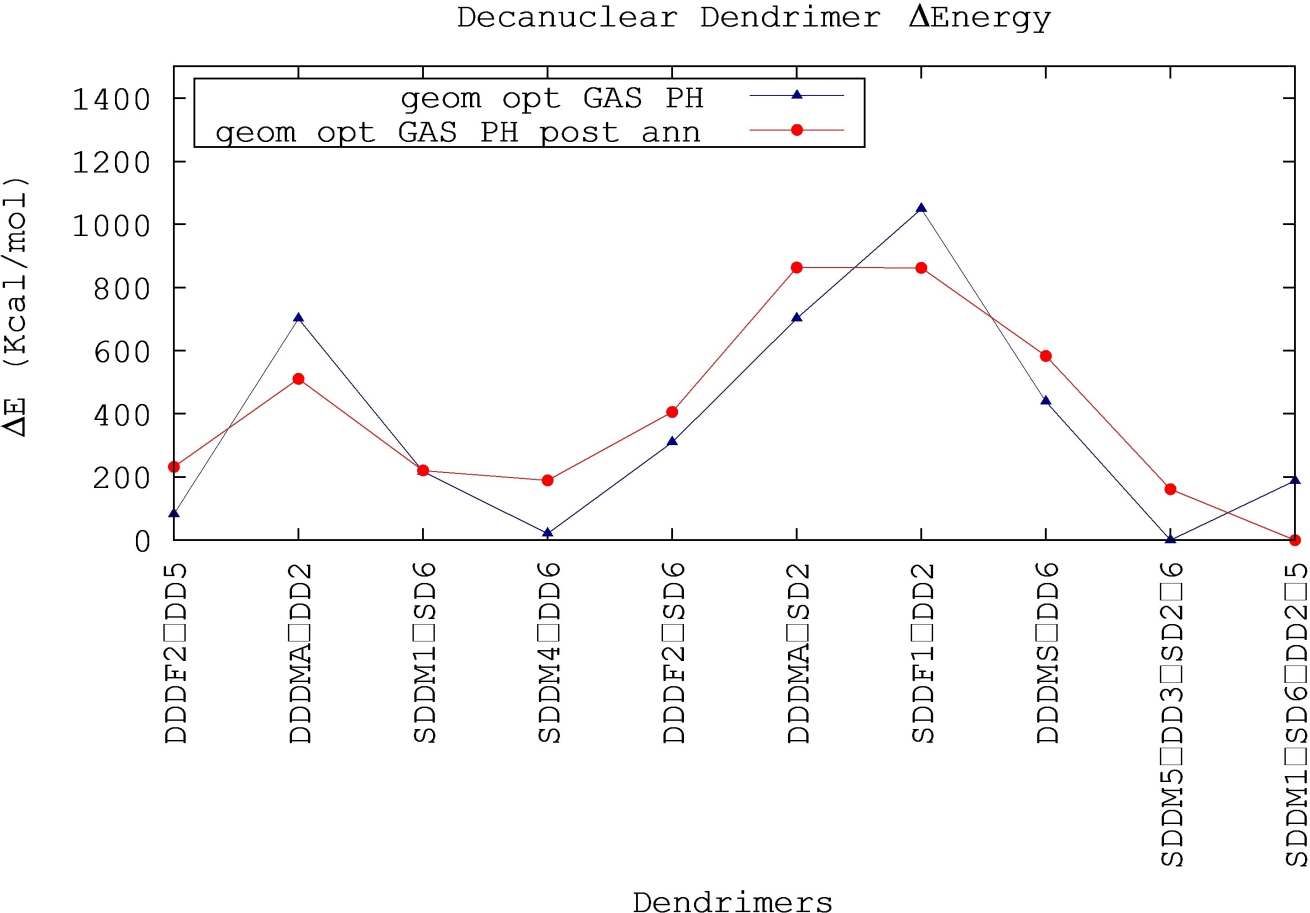
Relative stabilities (Δ*E*) of decanuclear dendrimers, obtained by subtracting the value of dendrimer with the lowest energy from each dendrimer energy. The Δ*E* values of the first geometry optimization run (blue line) and those of geometry optimization run after annealing procedure (red line) match almost perfectly, therefore the annealing run did not significantly alter the structure and stability of the complexes.

The relative energy difference ΔE values (in kcal/mol) of the decanuclear dendrimer models are shown in Figure 6. The relative energies after annealing are very close to the ones obtained before, indicating that the annealing procedure did not alter the structure and stability of the complexes.

It can be noted that the structures with a MER central core (in particular those with SDD ligands) are energetically favored (therefore more stable), according to experimental observations previously reported: in particular, NMR experiments on the complex [Ru(dpp)_3_]^2+^, which corresponds to the core of our dendrimers, have shown that the purified material is a mixture of the MER and FAC isomers in which the MER isomer predominates (92 %).^[29]^ In the case where the central core has a FAC structure, more stable dendrimers are obtained when the intermediate monomers have dpp ligands of the same type as those of the central monomer (ex: DDD central+DD intermediate).

Apparently, the stability of the dendrimers is not strictly linked to the energy of the individual monomers composing the dendrimer: for instance, the DDD FAC 02 (DD 05)_3_ dendrimer, which has the most stable core, presents a small minimization energy, but not the lowest. Similarly, the DDD MER SYM (DD 06)_3_ dendrimer, consisting of the isomers with highest ΔE, has not the highest overall energy. Overall, in order to obtain the dendrimers with lowest energy, one SDD MER central core plus three different intermediate monomers with alternated SD and DD ligands seems to be the better choice.

To further investigate the structure and the stability of the lowest energy dendrimers optimized in implicit solvent, we have carried out explicit solvent simulations of SDDMER01 DD02 SD06 DD05 dendrimer complex with PF_6_^−^ counterions in acetonitrile solution.

**Figure 7:**
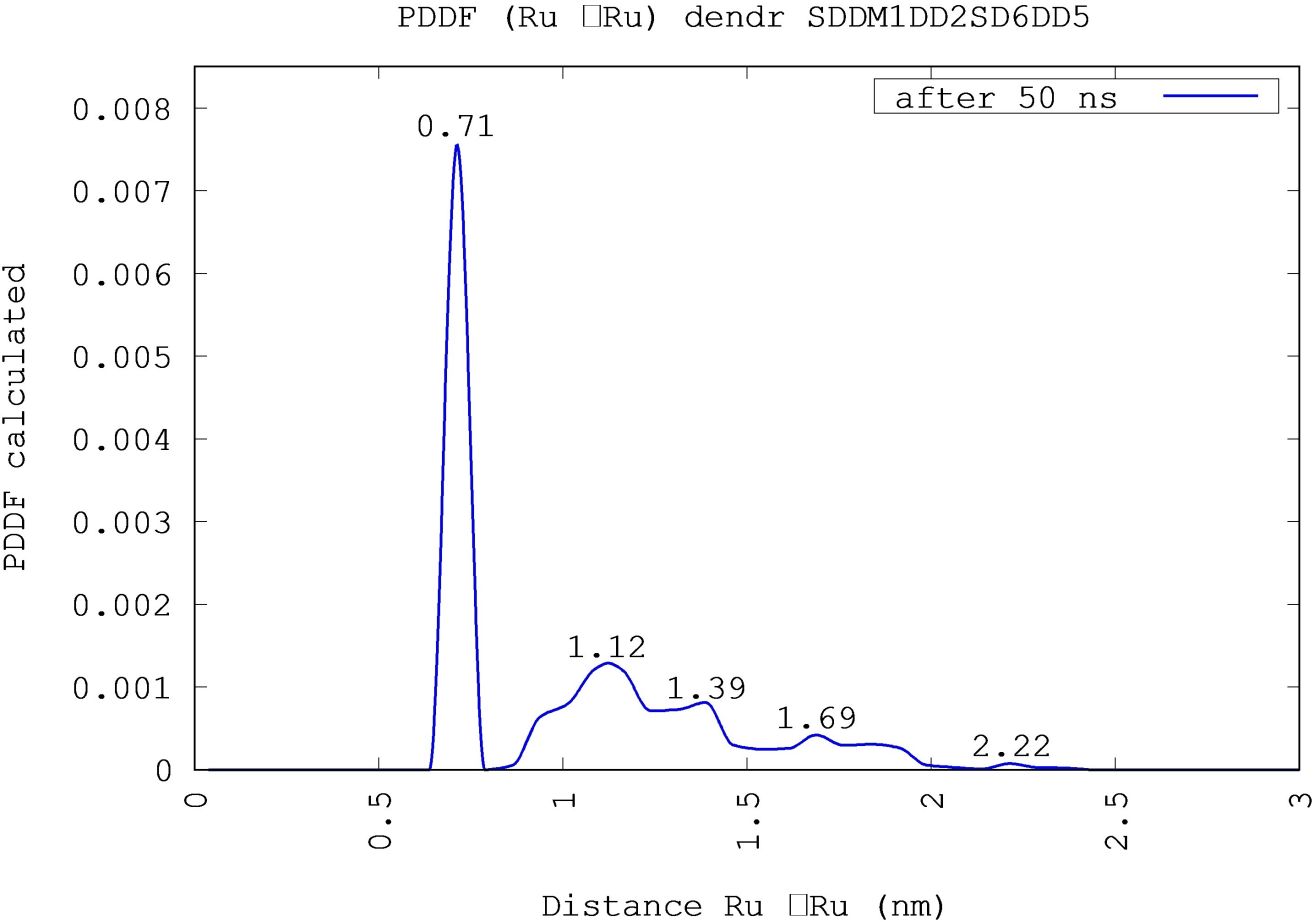
The distribution function of Ru−Ru pair distances concerning the SDDMER01+DD02+SD06+DD05 dendrimer shows the peak maxima corresponding, respectively, to values: 0.71 nm, 1.12 nm, 1.39 nm, 1.69 nm and 2.22 nm.

**Figure 8:**
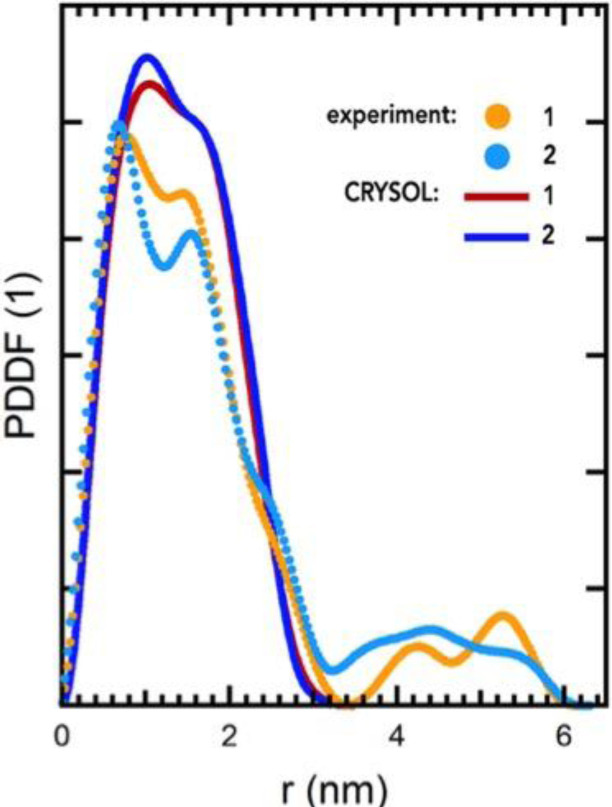
The Pair Distance Distribution Function experimentally obtained (orange and cyan line) by SAXS [16] on a sample of decanuclear dendrimers in acetonitrile solution with a concentration of 2.8 10^−5^ M. The orange line refers to experimental SAXS data for **OsRu9**, a decanuclear dendrimer with an osmium atom in the central unit and nine ruthenium centers in the intermediate and peripheral units; the cyan line refers to experimental SAXS data for **Ru10** (the compound investigated in our simulations; so it is this cyan line to be directly compared to the theoretical curve in Figure 7), and the red and blue lines refer to the theoretical spectra obtained from CRYSOL for **OsRu9** and **Ru10** dendrimers, respectively. The experimental curves show the peaks at 0.7 nm, 1.45 nm, 2.40 nm representing, respectively, the distance between central Ru and intermediate Ru, the distance between central Ru and peripheral Ru and the distance between two peripheral Ru atoms. Picture adapted from [16], Copyright licence provided by Elsevier and Copyright Clearance Center.

In order to investigate the dendrimer structure, we calculated the distribution function of Ru−Ru pair distances, shown in Figure 7, concerning the SDDMER01 DD02 SD06 DD05 dendrimer. Several peaks appears in the radial distribution function corresponding respectively to the values 0.71 nm, 1.12 nm, 1.39 nm, 1.69 nm and 2.22 nm. The first peak is due to the distance between the Ru atom of central monomer and the Ru atoms in the nearest monomers (i. e. the Ru atoms of intermediate monomers), the peaks at 1.12 nm and 1.39 nm come from the distance between the Ru atom of central core and the Ru atoms in the “second bracket” (i. e. the Ru atoms of peripheral cores), finally the peak at 1.69 nm and, more generally, the area up to 1.9 nm are involving distances between the more distant Ru atoms (i. e. peripheral Ru‐peripheral Ru); in addition the small signal at 2.22 nm describes the distance between the two most distant peripheral Ru atoms. This result agrees with the Pair Distance Distribution Function (PDDF) experimentally obtained by Small Angle X‐ray Scattering (SAXS) measurements as shown in Figure 8 (picture adapted from Ref. [16]). The PDDF spectrum in the case of Ru(II)‐based dendrimers, is dominated by high electron‐density clouds corresponding to the Ru(II) metal centers.^[16]^

**Figure 9:**
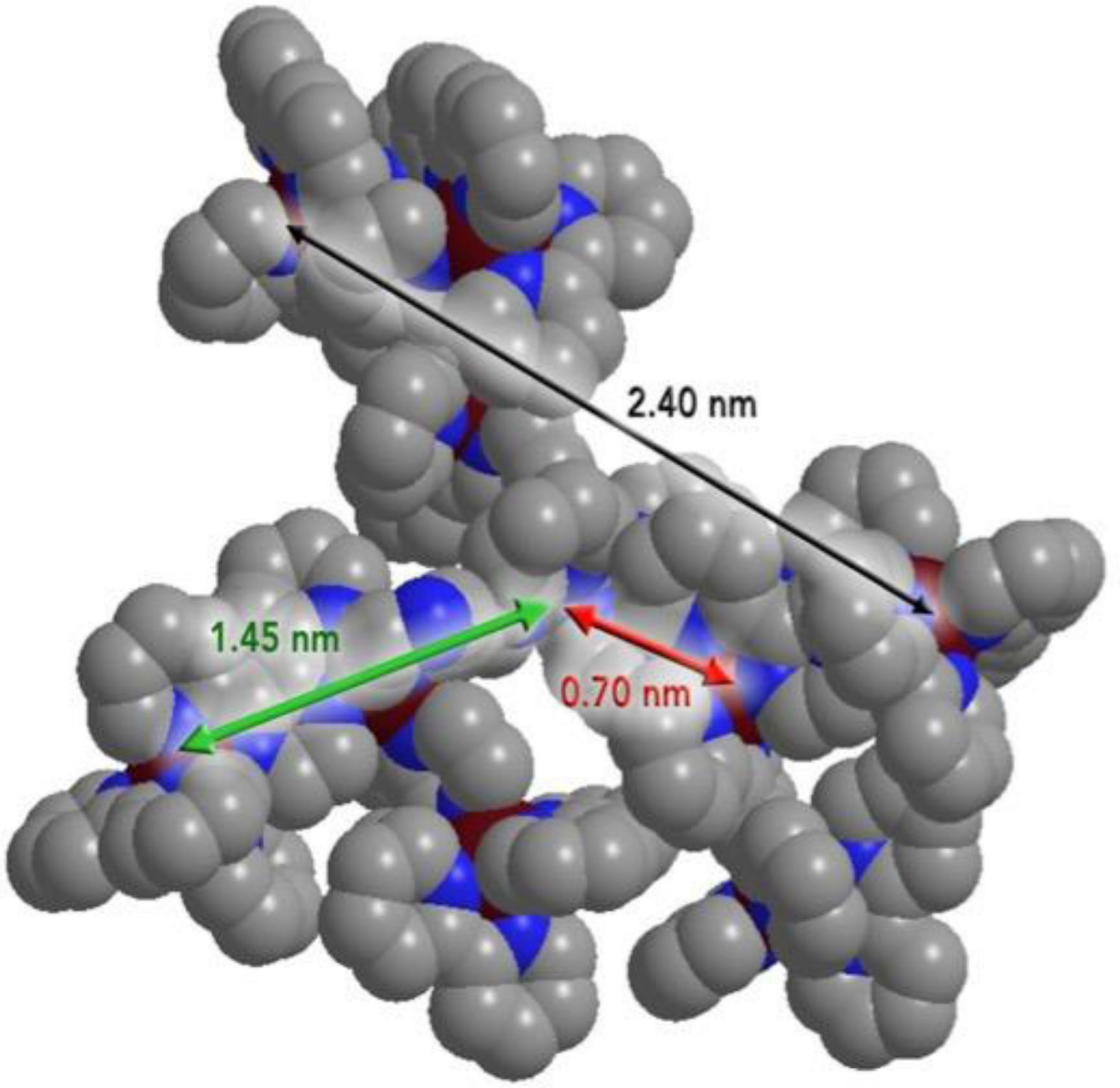
The distances between central Ru‐intermediate Ru (in red), central Ru‐peripheral Ru (in green) and peripheral Ru‐peripheral Ru (in black) obtained by PDDF using SAXS shown on dendrimer structure (picture from Ref. [16], Copyright licence provided by Elsevier and Copyright Clearance Center).

In Figure 8, the orange line refers to experimental SAXS data for **OsRu9**, a decanuclear dendrimer with an osmium atom in the central unit and nine ruthenium centers in the intermediate and peripheral units; the cyan line refers to experimental SAXS data for **Ru10** (the compound investigated in our simulations), and the red and blue lines refer to the theoretical spectra obtained from CRYSOL for **OsRu9** and **Ru10** dendrimers, respectively.^[16]^ The curves show peaks at 0.7 nm, 1.45 nm, 2.40 nm representing, respectively, the distance between central Ru and intermediate Ru, the distance between central Ru and peripheral Ru and the distance between two peripheral Ru atoms. These values, concerning an experimental sample, i. e. a mixture of isomers, suggested the structure shown in Figure 9.^[16]^

**Figure 10:**
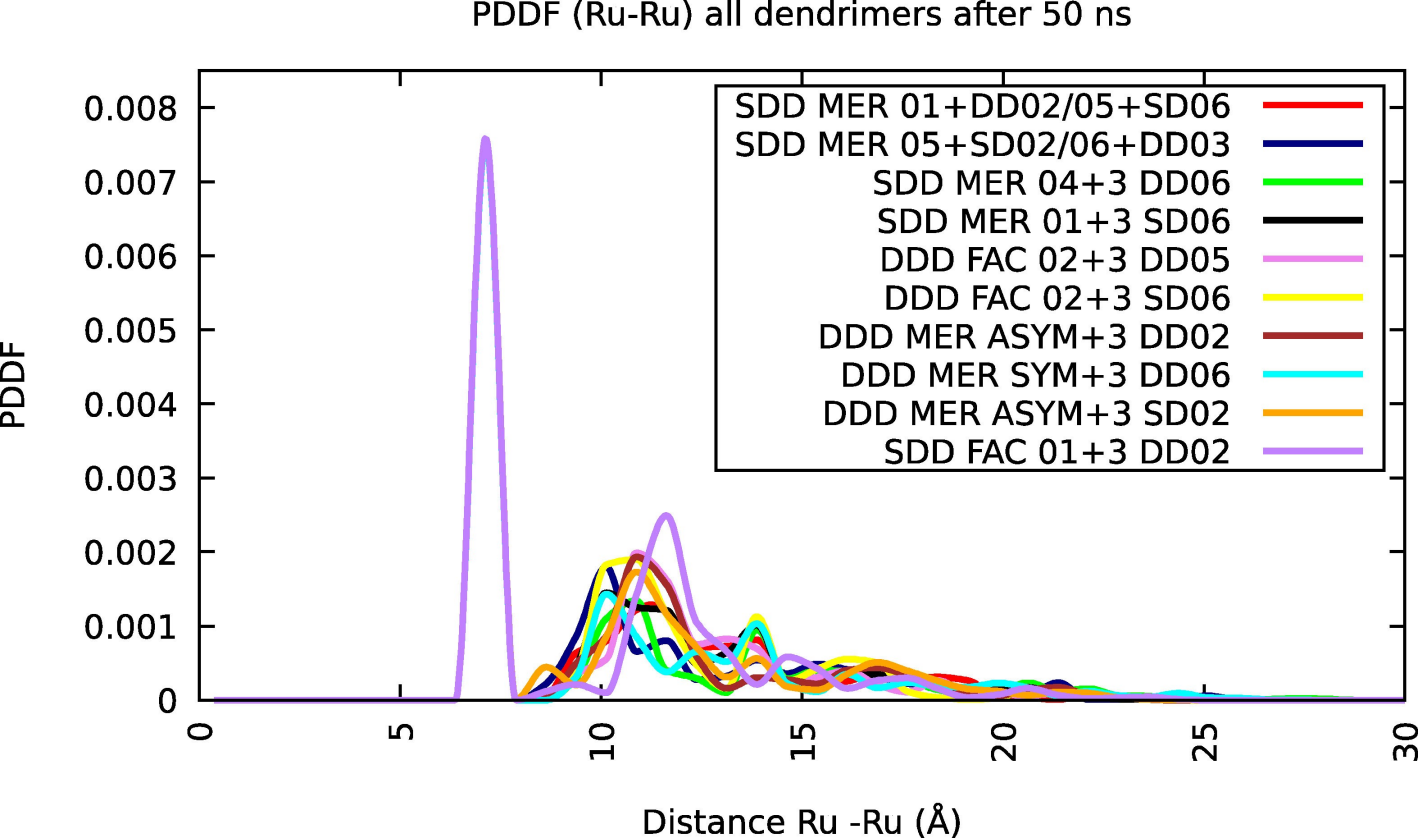
The PDDF of Ru−Ru distances of each dendrimer. The first peak is similar among all isomers and matches the experimental distance (this is not immediate from the color code); the subsequent peaks deviates in more extent among them and from experimental data since the peaks following the first, concerning the distance between central Ru‐peripheral Ru and peripheral Ru‐peripheral Ru, are largely influenced by the type of isomers (see details in Supporting Information). The corresponding experimental curve is the cyan curve in Figure 8.

We have also to keep in mind that the experimental PDDF obtained by SAXS refers to a mixture of isomers that self‐aggregate forming intermediate values of the Ru−Ru distances, whereas our data are referring to a single monomer model. Nevertheless, the first peak, obtained by simulation,^[16]^ is similar among all dendrimers here simulated (Figure 10) and matches the experimental distance; the subsequent peaks deviates in more extent among them and from the experimental data, since they are largely influenced by the type of isomers (see details in Supporting Information).

### Self‐aggregation of dendrimers

Despite their positive charge (each Ru(II) center has positive charge +2), the aggregation (self‐assembly) is a peculiar behaviour of dendrimers based on Ru and Os polypyridine complexes in acetonitrile solution, even at relatively low concentration (about 5×10^−5^ M).[^16^, ^31^] This is also interesting because the self‐assembly of light‐harvesting antenna systems is a typical feature of natural photosynthetic organisms, aimed at maximizing light absorption, energy transfer and solar energy conversion. Indeed, the experimental studies on Ru(II) (or Os(II)) based dendrimers showed that self‐assembly of light‐harvesting polypyridine complexes leads to aggregation‐induced energy transfer.^[16]^

To investigate the process of self‐assembling of different dendrimers we have selected two different modelling scenarios:

a system with 9 dendrimers, all different from each other, initially located faraway from each other
a system with 4 dendrimers, all of the same type (SDD MER 05+SD02+DD03+SD06) in a box with explicit acetonitrile solvent.

**Figure 11:**
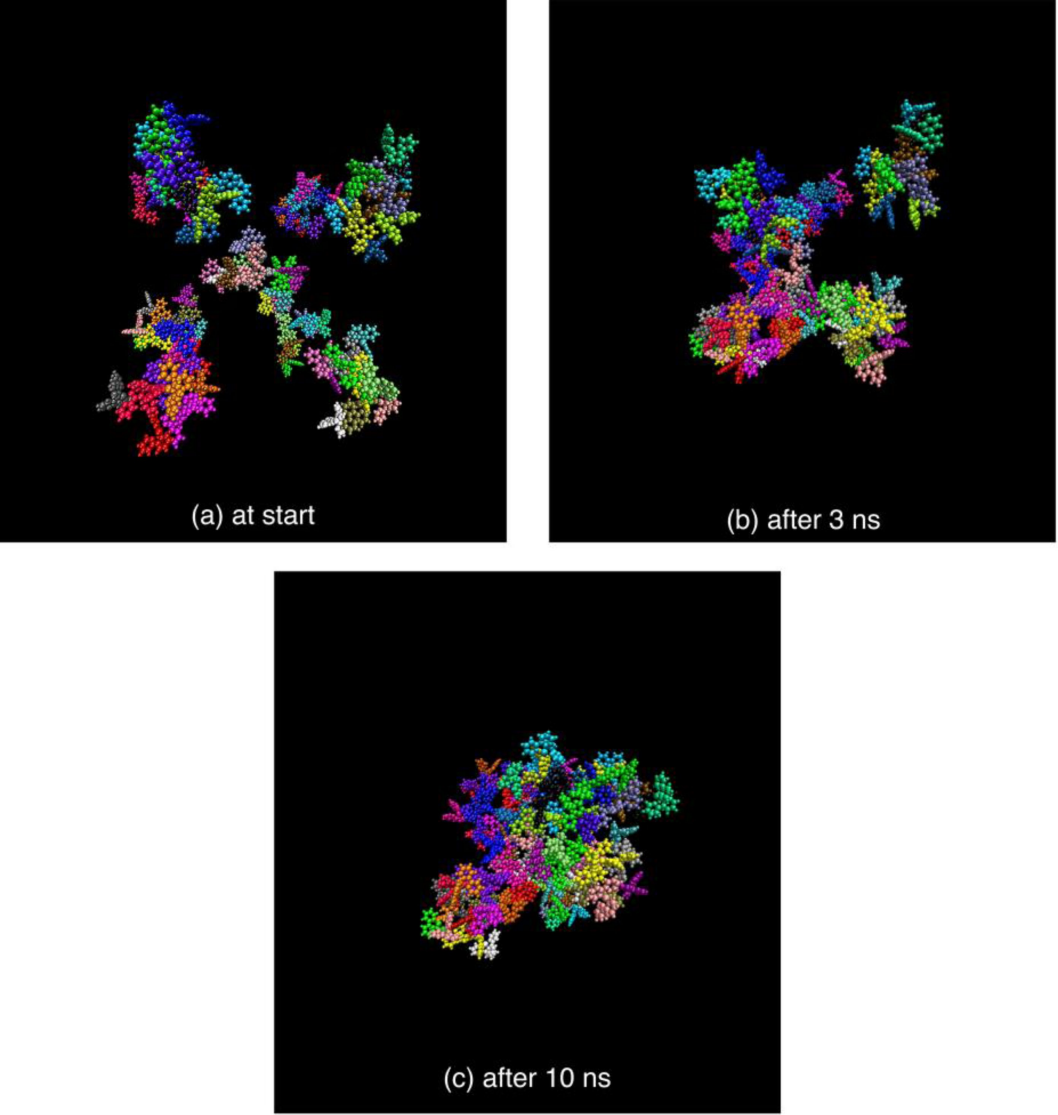
The snapshots of dendrimer position. Respectively: at start of simulations when 9 different dendrimers with random orientation are arranged in the cubic box with edge of 100 Å as far away as possible (a), after 3 ns when a group of dendrimers are aggregated (b) and finally after 10 ns when all the dendrimers are aggregated (c). NB: The PF_6_^−^ molecule are not displayed.

Both systems upon dynamics arrive to a more compact geometry, leading to the aggregation of the dendrimer polymers, as shown in the snapshots of Figure 11, which shows the respective positions of dendrimers at start of simulations, after 3 ns and after 10 ns. We can observe that after 3 ns a group of dendrimers aggregates, finally after 10 ns all the dendrimers are aggregated.

**Figure 12:**
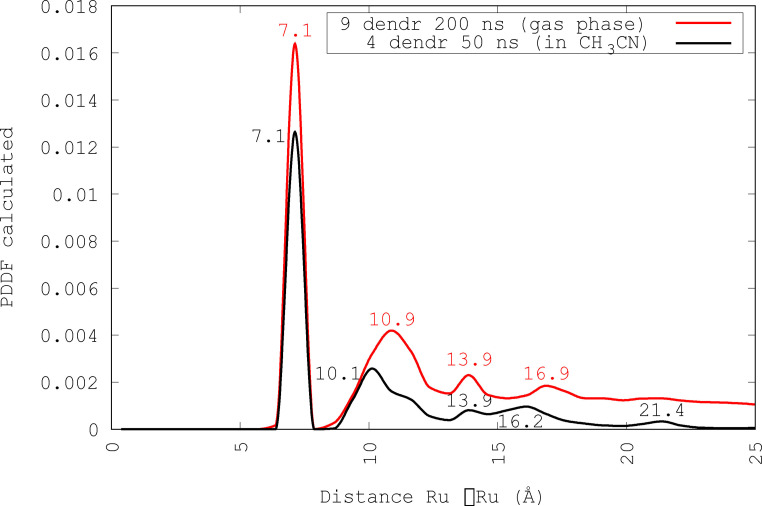
The distribution function of the Ru−Ru distances, after 200 ns, obtained from MD simulations on nine different dendrimers with random orientation in gas phase (red line) and the same function after 50 ns, obtained from MD simulations on four identical dendrimers in acetonitrile solution (black line). With regard to nine dendrimers, we can observe some peaks, respectively, at 7.1 Å, 10.9 Å, 13.9 Å and 16.9 Å. With regard to four dendrimers, the peaks at 7.1 Å, 10.1 Å, 13.9 Å, 16.2 Å and 21.4 Å are obvious. All the peaks match with the experimental peaks obtained by SAXS: 7.0 Å, 14.5 Å and 24.0 Å.

By analizing the distribution function of Ru−Ru distances related to the first case after 200 ns, shown in Figure 12 (red line), we can observe peaks respectively at 7.1 Å, 10.9 Å, 13.9 Å and 16.9 Å.

Table 1 shows a very good match between the experimental peaks, the mean value of peaks concerning a single dendrimer after 50 ns and the nine aggregated dendrimer peaks. Probably the differences in the end zone of PDDF and the absence of peak II (between experimental and MD simulation data) depend on different component isomers (the experimental sample contains a not monitoring mixture of isomers while the simulated sample contains only the selected isomers) that have rings more or less close, so the Ru(II) centers are arranged more or less far.

**Table 1:** Comparison between the values (in Å) of PDDF peaks. The first row contains the experimental results using SAXS, the second row contains the results of MD simulations on the most stable dendrimer: SDDMER01+DD02+SD06+DD05; the average values of the peaks obtained from the MD simulations on all single dendrimers are in the third row, the fourth row shows the values concerning nine different aggregated dendrimers and finally the fifth row shows the values concerning four dendrimers all the same. The peak II is completely missing in the experimental Figure 8; in fact, the distances between peripheral Ru atoms belonging to “facing branches” contribute mostly to this peak.

	I	II	III	End region
Exp PDDF	7.0		14.5	24.0
SDDM1DD2SD6DD5	7.1	11.2	13.9	16.9–22.2
mean value sing. dendr	7.1	11.0	13.8	16.6–21.7
9 dendr. gas phase	7.1	10.9	13.9	16.9–24.4
4 dendr acetonitr. solution	7.1	10.1	13.9	16.9–21.4

**Figure 13:**
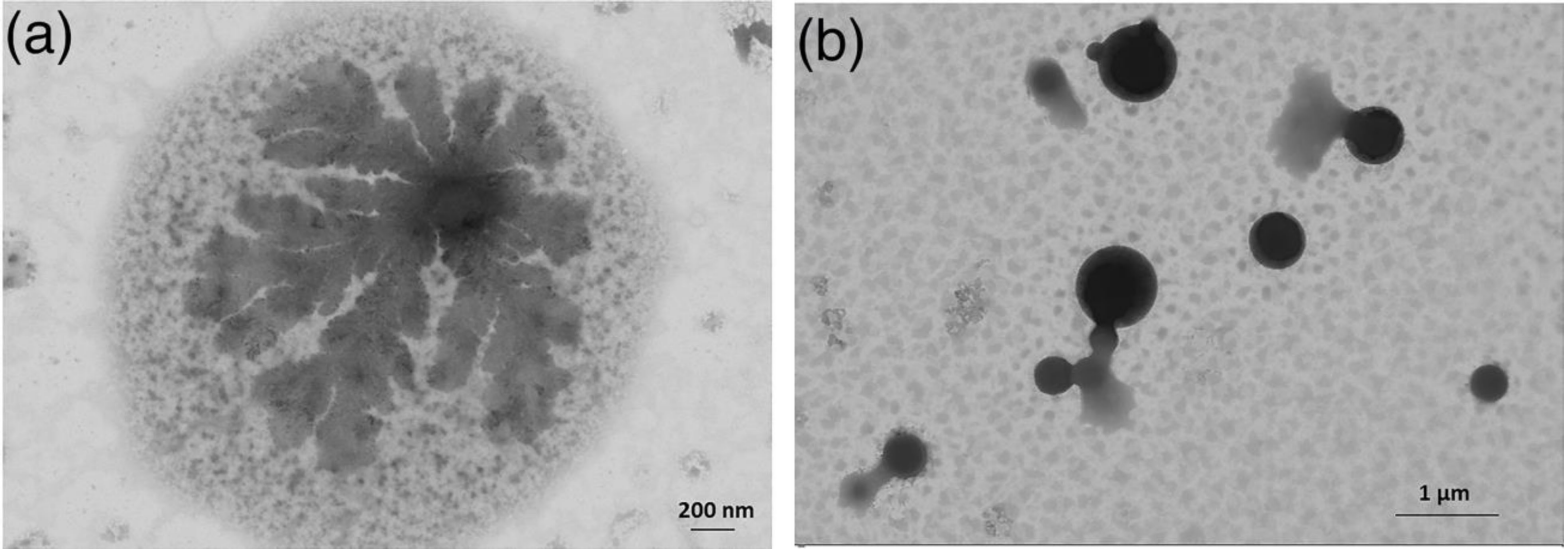
Panel (a): STEM image obtained from diluited (5×10^−6^ m) acetonitrile solution of **Ru10**. Panel (b): STEM image obtained from concentrated (5×10^−5^ m) acetonitrile solution of **Ru10**.

From the above‐mentioned results, it is suggested that aggregation process takes place in at least two steps: the first step involves aggregation of a discrete number (2–3) of dendrimers, and it is followed by aggregation of pre‐aggregated smaller systems into larger entities. The above proposed mechanism of aggregation is confirmed by scanning transmission electron microscopy (STEM) experiments. Actually, STEM experiments on **Ru10** have been made using different concentration in acetonitrile solutions. A drop of **Ru10** acetonitrile solutions with concentration lower than 5 x10^−6^ m, deposited on gold surfaces and upon solvent evaporation, yielded images of domains of fractal‐type aggregates (Figure 13a). The composition of the system was determined by energy‐dispersive X‐ray (EDX) analysis at different points of the sample, see Figure 10 in Supporting Information. The estimated C (on average, 60 %), O (on average, 11 %), F (on average, 2.0 %), P (on average, 1.4 %) and Ru (on average 0.8 %) percentages vary slightly from one point of the sample to another point, confirming the nature of such fractal‐type aggregates as made of **Ru10** compounds. The fractal‐type aggregates coalesce into much larger – and possibly more compact – aggregates, with dimensions between 25–100 microns, when the solution of **Ru10** is increased above 5×10^−5^ m (Figure 13b).

In this case, as evidenced by the EDX analysis (see Figure 11 in the Supporting Information). the percentage of F is slightly higher (about 4 %) with respect to the less concentrated sample, the percentage of P remains substantially unchanged (about 1 %) and that of the Ru is, on average, of about 0.7 %.

With regard to the position of PF_6_^−^ counteranions, the distribution function of distances between ruthenium centers and PF_6_^−^ anions shows, as in the case of a single dendrimer, that the anions arrange near the dendrimer molecules. This can be observed in PDDF graph (Figure 9 of the Supporting Information) showing the distribution function of distances between Ru(II) centers and PF_6_^−^ anions after 200 ns. Peaks at 0.61 nm, 1.08 nm, 1.51 nm and 3.6 nm are evident. These peaks show that most of PF_6_^−^ anions are arranged as close as possible to Ru atoms (i. e. 0.61 nm considering the presence of ligands).

The case of four dendrimers (SDD MER 05+SD02+DD03+SD06), in acetonitrile solution is briefly reported, because it is very similar to the case of the aggregation of the nine dendrimers previously discussed. We analyzed the distribution function of Ru−Ru distances after 50 ns, shown in Figure 12 (black line), and five peaks respectively at 0.71 nm, 1.01 nm, 1.39 nm, 1.62 nm and 2.14 nm are evident. These peaks match with the peaks obtained from the distribution function of the Ru−Ru distances in MD simulations in implicit solvent (cfr. Table 1). In that case, the peaks were at 0.71 nm, 1.09 nm, 1.39 nm and 1.69 nm, with a little “hump” at 2.44 nm. Moreover, they also fairly match the experimental distances obtained by SAXS (0.70 nm, 1.45 nm and 2.40 nm).^[16]^

The distribution of the distance Ru^2+^−PF_6_^−^ is also absolutely similar to that obtained in the case of the nine dendrimers aggregation in implicit solvent. In the case of the four dendrimers, in explicit solvent we obtained three peaks at 0.57 nm, 0.94 nm and 1.56 nm. These peaks roughly match the corresponding peaks in the case of nine different dendrimers in implicit solvent: 0.61 nm, 1.08 nm, 1.51 nm. Overall, since the behavior of dendrimers in simulations containing acetonitrile molecules is absolutely similar to that in implicit solvent simulations, we can infer that the implicit solvent model provides a good approximation of the molecules behavior during the aggregation process.

## Conclusions

In this work we presented the first attempt to computationally tackle the structure of light‐harvesting metal polypyridine dendrimers, already known to be involved in photoinduced water oxidation processes relevant for artificial photosynthesis. Focus is here on a representative compound, **Ru10**. To allow to face a very complex problem, most of the work has been focussed on geometrical isomerisms (including MER and FAC core isomers, plus stability of D and S “conformers”, which are expected to have significant roles in the overall structures of the decanuclear dendrimers), leaving aside the Δ and Λ stereoisomerism of each metal building block.

Thanks to good agreement between QM and MD simulations (with force field properly reparameterized) the number of stable building block isomers is reduced to 36, but the number of possible combinations in a decanuclear dendrimer still remains high (of the order of 10^6^); therefore, particular dendrimers have been composed and investigated in search of some more general rule.

The energies obtained by geometry optimization suggest that:

the structures with a MER central core (in particular those with SDD ligands) are energetically favored, according to the experimental NMR observations;^[29]^
if the central core has a FAC structure, more stable dendrimers are obtained when the intermediate monomers have dpp ligands of the same type as those of the central monomer (e. g.: DDD central+DD intermediate);
to obtain the dendrimers with lowest energy, one SDD MER central core plus three different intermediate monomers with alternated SD and DD ligands has proven to be a good choice;
the stability of the dendrimer is not strictly linked to the energy of the individual monomers composing the dendrimer.

Wa have also faced the self aggregation of **Ru10**. On this regard, MD simulations, in acetonitrile solution and with PF_6_^−^ counterions shows that the distances between the Ru atoms in the calculated structure of dendrimers, calculated through the pair distance distribution function, reflect those experimentally obtained by the SAXS.

Finally, some groups of dendrimers were investigated both in gas phase and in acetonitrile solution. In all cases, both the movies of trajectories and the calculated pair distance distribution function (for the Ruthenium pairs) confirm the aggregation between the dendrimers and suggest its mechanism: the dendrimers are approaching in small blocks and then aggregate all together.

Summarizing, our results show the succesfully application of computational methods to investigate the structure, as well as the self‐aggregation behavior, of quite large photo‐ and redox‐active artificial metal‐based dendrimers, although limiting the study, in this first attempt, to geometrical isomers and neglecting stereoisomers. Since smaller dendrimers aggregate with molecular water oxidation catalysts to give arise to functional systems for artificial photosynthesis,[^14c^, ^15^] our results can open the way towards integrated antenna‐catalysts assemblies which promise to hold great relevance for potential solar energy conversion.

## Supporting Information available

Contains details on the computational aspects of the study and STEM/EDX experiments: 11 figures and 2 tables).

## Conflict of interest

The authors declare no conflict of interest.

## Supporting information

As a service to our authors and readers, this journal provides supporting information supplied by the authors. Such materials are peer reviewed and may be re‐organized for online delivery, but are not copy‐edited or typeset. Technical support issues arising from supporting information (other than missing files) should be addressed to the authors.

Supporting Information
