# Supplementary material for: Molecular Modelling and Simulations of Light‐Harvesting Decanuclear Ru‐Based Dendrimers for Artificial Photosynthesis
Source: Chemistry. 2021 Dec 16;28(5):e202103310. doi: 10.1002/chem.202103310 (PMC9299829; doi:10.1002/chem.202103310)
Supplement: Supplementary file 1 — Supporting Information [file CHEM-28-0-s001.pdf]

Chemistry–A European Journal

Supporting Information

Molecular Modelling and Simulations of Light-Harvesting Decanuclear Ru-Based Dendrimers for Artificial Photosynthesis

Giovanna M. A. Rogati, Chiara Capecci, Enza Fazio, Scolastica Serroni, Fausto Puntoriero, Sebastiano Campagna,* and Leonardo Guidoni*

Table of contents *Pages*

Computational Part ***3***

MD Simulations (Computational Details) ***3***

Table 1 3

Force Field Reparameterization (Detailed Procedure) ***3***

Figure 1. *4*

Figure 2. *4*

Figure 3. *5*

Figure 4. *6*

Figure 5. *7*

Table 2. *7*

Further considerations on the computational part ***8***

i. **Monomers/building blocks** 8

Figure 6 *8*

ii. **Investigating the dendrimer structure** **9**

Table 3 *9*

Figure 7 *10*

Figure 8 10

iii. **Self-aggregation** **11**

Figure 9 *11*

Experimental details related to STEM experiments **12**

Figure 10 *12*

Figure 11 *13*

References and Notes **14**

Computational Part

MD Simulations (Computational Details)

The ruthenium(II) polypyridyl complex was represented using parameters from the AMBER/parm99 force field, supplemented by the parameters developed by Norrby et al. [1] for ruthenium polypyridyl compounds, to which appropriate unit corrections were applied.[2,3] The additional parameters used in the simulation are listed in Table 1.

Table 1. The additional force-field parameters for ruthenium polypyridyl compounds. The symbols in the first column refer to the standard AMBER nomenclature [4]

atoms	parameters	ref
Ru	R_{vdW} = 2.963 Å<br>ε = 0.56 kcal•mol^{-1}	[3]
Ru-N	r_0 = 2.081 Å<br>K_{bond} = 268 kcal•mol^{-1}•Å^{-1}	[1]
Ru-N-CA	θ_0 = 123.5°<br>K_{ang} = 103.6 kcal•mol^{-1}•rad^{-1}	[1]
N-Ru-N (cis)	θ_0 = 91.1°<br>K_{ang} = 81.25 kcal•mol^{-1}•rad^{-1}	[1]
N-Ru-N (trans)	θ_0 = 180.0°<br>K_{ang} = 24.49 kcal•mol^{-1}•rad^{-1}	[1]
Ru-N-CA-CA	V_N / 2 = 1.21 kcal•mol^{-1}<br>γ = 0° n = -2	[1]
Ru-N-CA-CA	V_N / 2 = 1.41 kcal•mol^{-1}<br>γ = 180° n = 1	[1]
Ru-N-CA-HA	V_N / 2 = 5.27 kcal•mol^{-1}<br>γ = 180° n = 2	[1]
N-Ru-N-CA	V_N / 2 = 0.25 kcal•mol^{-1}<br>γ = 180° n = 4	[1]

Force Field Reparameterization (Detailed Procedure)

Observing the MD optimized structures of monomers, we noticed that some medium rings of the dpp were distorted as shown in **Figure 1** obtained by VMD.[5] For this reason, the force field (FF) related to the dihedral angle N-C-C-N has been reparameterized.

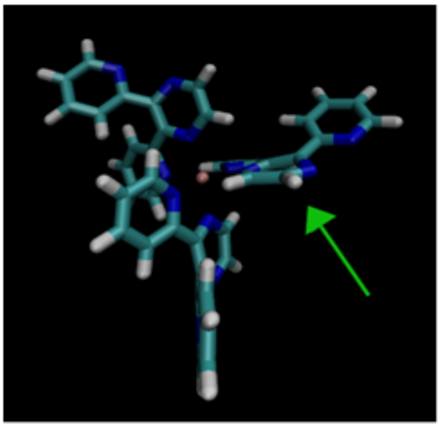

Figure 1. An example of a distorted ring. The blue vertices represent the nitrogen atoms, the cyan ones the carbon atoms, the white ones the hydrogen atoms, and finally the pink one the ruthenium atoms. This distortion is due to the chosen parameters of the force field, in particular because of those related to the dihedral angle N-C-C-N.

In order to reparameterize the FF of dihedral angle N-C-C-N (and consequently the C-C-C-C and N-C-C-C dihedrals including the bond between pyridyl rings) shown in **Figure 2**, we performed QM simulation with relaxed surface scan,[6] in which we could scan through one variable (in our case the C-C-C-C dihedral angle) while all the others were relaxed, on a bipyridine molecule.

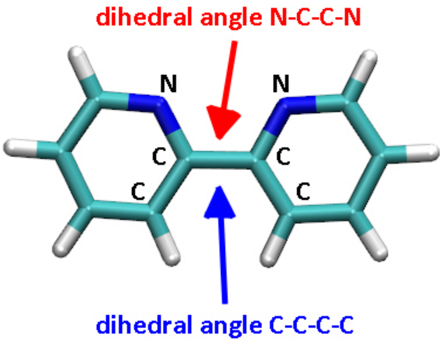

Figure 2. The dihedral angle N-C-C-N and its explementary C-C-C-C.

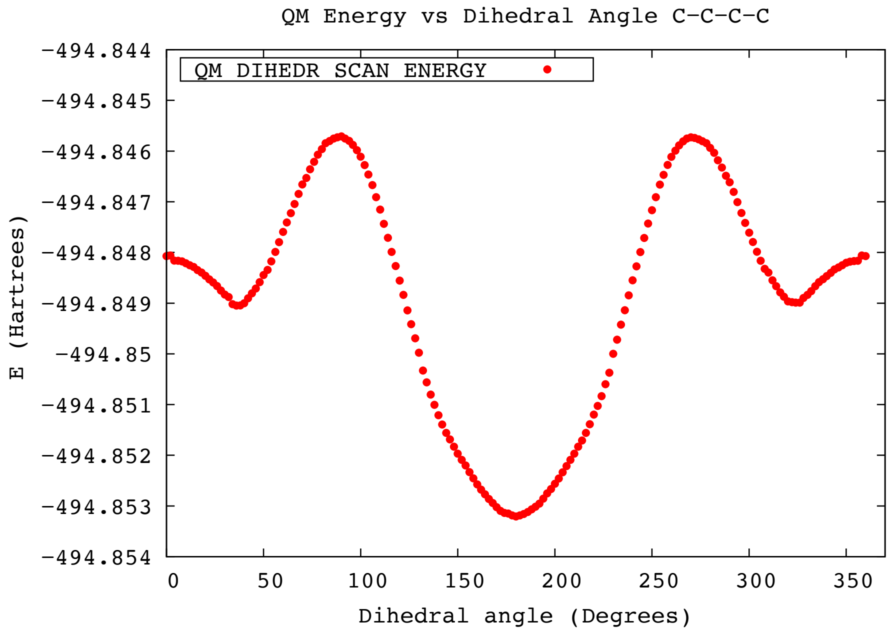

Figure 3. The data (red points) represent the results of QM dihedral scan simulations on bipyridine. Three minima are evident at 37.1°, 180.3° and 322.6° (respectively, 0.65 rad, 3.15 rad and 5.63 rad).

We have scanned through dihedral angle C-C-C-C from 0 to 360° in 181 steps (2°). We obtained 3 minima at 37.1, 180.3, and 322.6° (respectively, 0.65 rad, 3.15 rad and 5.63 rad) as shown in **Figure 3**. This behaviour is confirmed by literature:[7] an analysis of the torsional profile for bpy with different model chemistries and basis sets shows a strong stabilization of the coplanar nitrogen anti conformation (global minimum at 180°) and a second minimum for the cis conformation with a dihedral of about 40° (local minimum in B3LYP electronic energy curve at 39.5°). Then, we performed MD simulations, with scan on dihedral angle we are interested in, but with C-C-C-C dihedral force constant reset to zero. **Figure 4** illustrates the trend of energy vs dihedral angle.

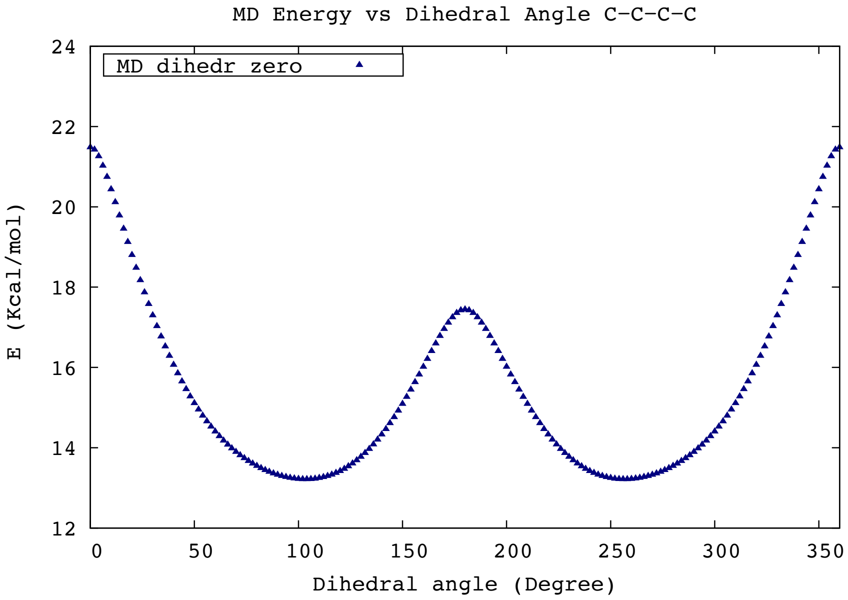

Figure 4. Energy vs dihedral angle obtained by MD simulations (with scan on dihedral angle) with C-C-C-C dihedral force constant reset to zero.

In order to obtain the torsion angle parameters, we subtracted the results of the QM simulations to those of the MD simulations:

$$E_{dihedr\ C-C-C-C} = E^{QM}_{scan\ diedr} - E^{MD}_{diedr\ zero}$$

The Energy difference $E^{QM}_{scan\ diedr} - E^{MD}_{diedr\ zero}$ represents the potential energy due to the torsion.[8]

Finally, the data are fitted to Fourier series:

$$f(t) = \frac{a_0}{2} + \sum_{n=1}^{N} [a_n \cdot \cos(nt) + b_n \cdot \sin(nt)]$$

A good fit was obtained with a series expansion until the fourth term, but we have chosen to expand the series up to the twelfth term to get a better match. The fit was performed by MATLAB and Statistics Toolbox Release 2014a.[9] **Figure 5** shows the data ($E^{QM}_{scan\ diedr} - E^{MD}_{diedr\ zero}$) and the fit. The results of fitting are shown in **Table 2** which also contains the new parameter values (appropriately derived as reported in ref. 10).

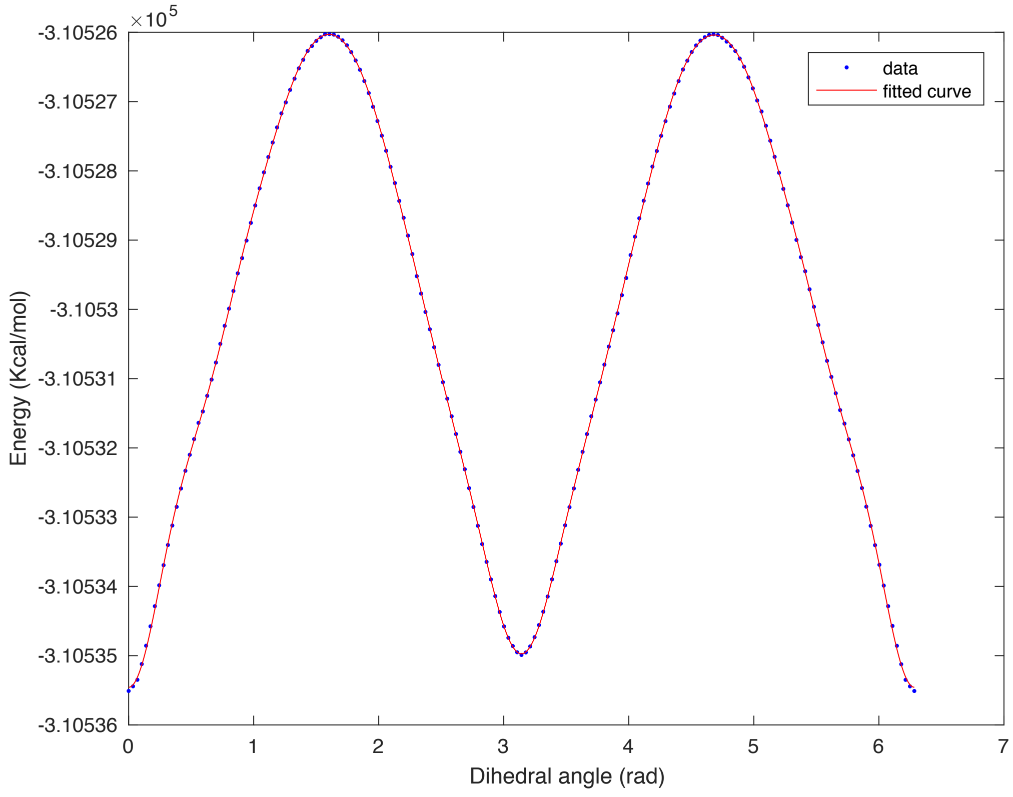

Figure 5. The fit to obtain the new parameter value for dihedral angle C-C-C-C (and for dihedrals related to it): the data (blue points) represent $E^{QM}{}_{scan\ diedr} - E^{MD}{}_{diedr\ zero}$, the curve (red line) is the Fourier series expansion up to the twelfth term.

Table 2. Fitted parameters for the C-C-C-C dihedral angle. The results of fit in the first two columns and the new parameters of dihedral angle in the third and fourth column. The a_n, b_n and V_n values are in kcal•mol^{-1}; the Φ_n values are in degrees.

a_1 = -0.1282	b_1 = 0.0162	V_1 = 0.128	Φ_1 = 180.930
a_2 = -4.235	b_2 = 0.0012	V_2 = 4.235	Φ_2 = 180.068
a_3 = 0.0588	b_3 = -0.0092	V_3 = 0.059	Φ_3 = 359.471
a_4 = -0.2842	b_4 = -0.007	V_4 = 0.284	Φ_4 = 179.598
a_5 = -0.0740	b_5 = -0.0069	V_5 = 0.074	Φ_5 = 179.605
a_6 = -0.281	b_6= -0.0068	V_6= 0.281	Φ_6 = 179.611
a_7 = -0.0378	b_7 = 0.0139	V_7 = 0.038	Φ_7 = 180.798
a_8 = -0.1494	b_8 = -0.0112	V_8 = 0.149	Φ_8 = 179.358
a_9 = -0.0375	b_9 = -0.0348	V_9 = 0.038	Φ_9 = 178.002
a_{10} = -0.0707	b_{10} = 0.0299	V_{10} = 0.071	Φ_{10} = 181.713
a_{11} = -0.0235	b_{11} = 0.137	V_{11} = 0.023	Φ_{11} = 187.850
a_{12} = -0.0281	b_{12} = 0.0226	V_{12} = 0.028	Φ_{12} = 181.296

Further considerations on the computational part

i. Monomers/building blocks

Observing the MD and QM optimized structures of monomers, we noticed that the nitrogen atoms of the free chelating site (in our case, the chelating site consists of two nitrogen atoms of two different rings from the same dpp that are donor atoms for attachment to another monomer metal. In the case of dendrimers, each metal is coordinated by three chelators) were arranged at the antipodes in some monomers, as shown in **Figure 6**. These isomers (actually, conformers) have been excluded, because this conformation prevents the creation of a bond with ruthenium to form multinuclear complexes.

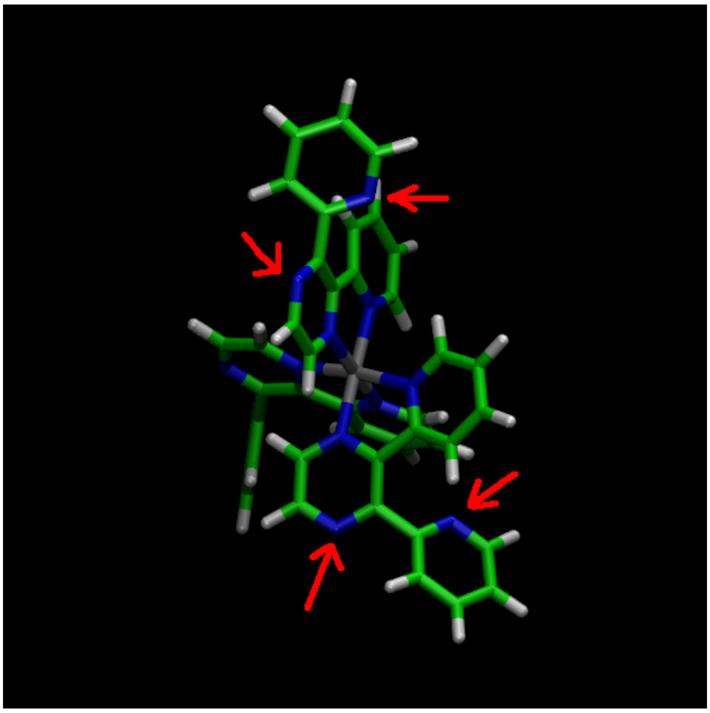

Figure 6. Example of a momoner discarded because the nitrogen atoms of chelating site (highlighted with red arrow) occupy opposite positions (dihedral angle N-C-C-N ∿ 180°). This condition would prevent the creation of a connection with ruthenium to form the dendrimer molecule.

ii. **Investigating the dendrimer structure**

The following **Table 3** shows the value in Å of peaks obtained after 50 ns of MD simulation in acetonitrile solution on each dendrimer. For the peaks of each dendrimer, the same considerations reported in the main article apply. PF_6^- counterions, initially distributed randomly around the dendrimer, moved close to Ru ions to neutralize their positive charges. By observing the snapshots of the trajectories (see **Figure 7**, concerning the SDDMER01+DD02+SD06+DD05 dendrimer), it can be noted that after just 1 ns the ions are placed at the minimum allowed distance from the Ru ions to neutralize their positive charges. The acetonitrile molecules, present in the simulation box, are not displayed in pictures.

Table 3. Positions of the peaks of the PDDF obtained after 50 ns of MD simulation for each dendrimer expressed in Å.
The numbers in the first column identify the dendimer type according to the following correspondence: ①=SDD MER 01 + DD02+SD06+DD05, ②= SDD MER 05 + SD02+SD06+DD03,③=SDD MER 04 + 3 DD06, ④=SDD MER 01 +3 SD06, ⑤=DDD FAC 02 + 3 DD05, ⑥=DDD FAC 02 + 3 SD06, ⑦=DDD MER ASYM + 3 DD02, ⑧=DDD MER SYM+ 3 DD06, ⑨=DDD MER ASYM + 3 SD02, ⑩=SDD FAC 01 + 3 DD02. Peck I represents the distance between central Ru-intermediate Ru; Peaks II and III are in the region where distances between central Ru and peripheral Ru are found; Peacks IV and V correspond to distance between peripheral Ru-peripheral Ru.

Dendrimer	Peak I	Peak II	Peak III	Peak IV	Peak V	(Å)
①	7.1	11.2	13.9	16.9	22.2	
②	7.1	11.2	13.9	16.9	22.2	
③	7.1	10.8	13.9	15.6	20.7-22.1	
④	7.1	10.2	13.9	16.5	20.6	
⑤	7.1	11.1	13.2	16.2	18.4-21.4	
⑥	7.1	11.0	13.9	16.5	20.7	
⑦	7.1	11.1	14.0	16.7	21.4	
⑧	7.1	11.2	13.9	16.9	22.2	
⑨	7.1	11.2	13.9	16.9	22.2	
⑩	7.1	11.6	13.9	16.9	22.2	

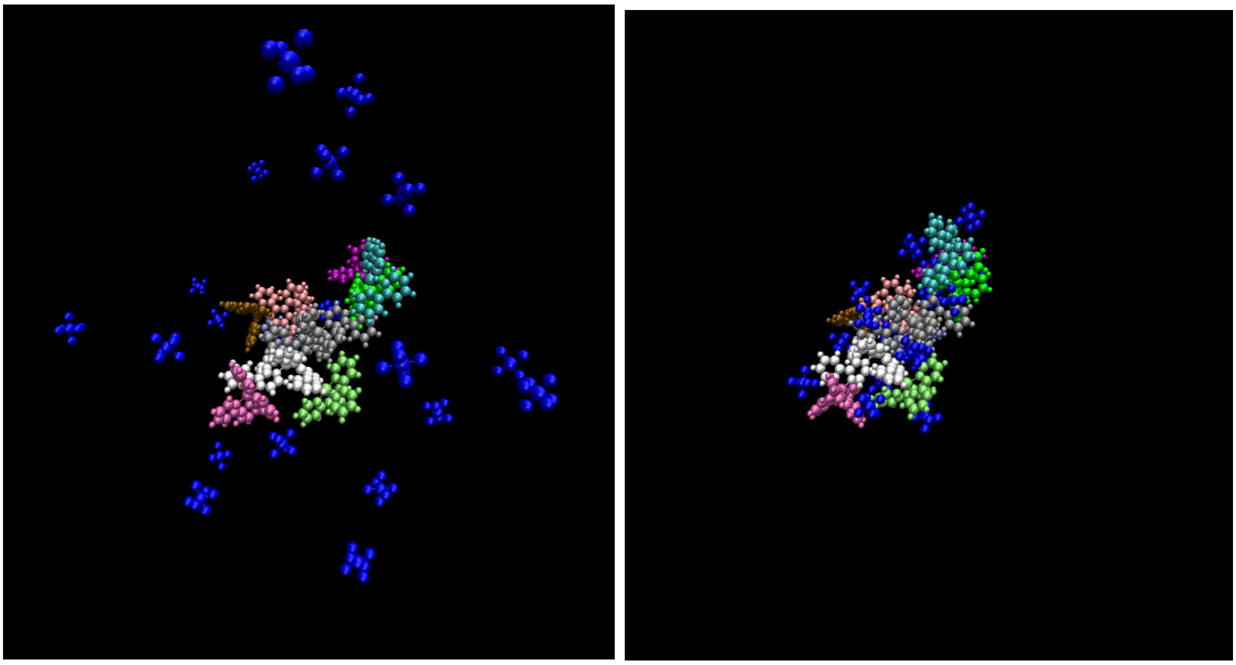

Figure 7. The snapshots of position of PF_6^- counterions (blue molecules) around the SDDMER01+DD02+SD06+DD05 dendrimer during the MD simulation. Initially (left), the PF_6^- anions are distributeded randomly, around the dendrimer; after just 1 ns (right) the ions are placed at the minimum allowed distance from the Ru ions to neutralize their positive charges. NB: the acetonitrile molecules, present in the simulation box, are not displayed in pictures.

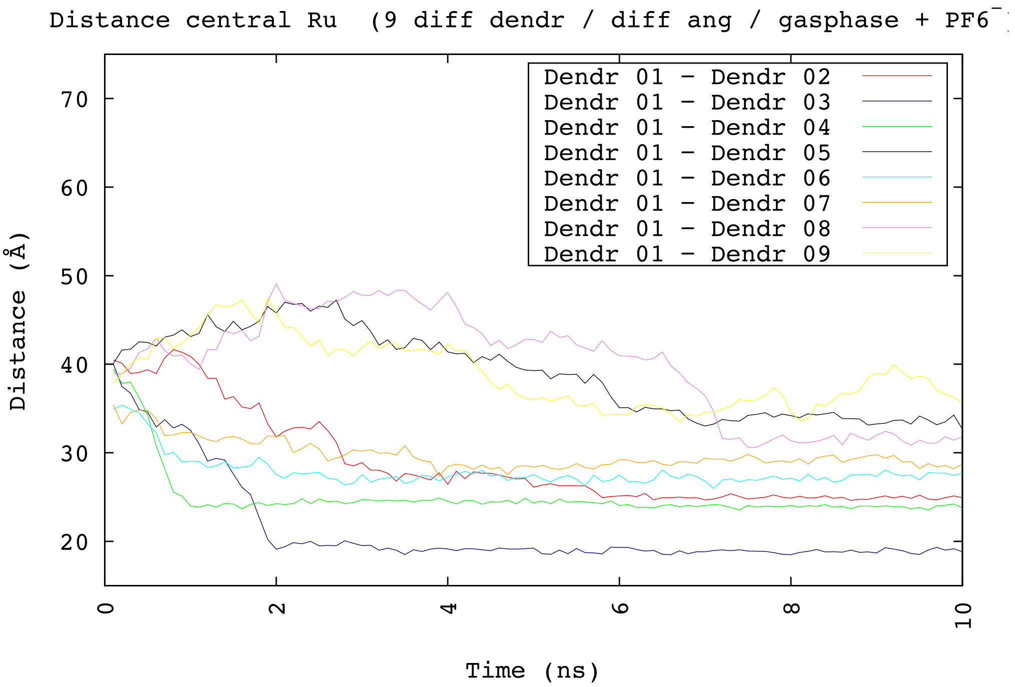

Figure 8. The distances vs time between the central Ru atom of dendrimer 01 and the central Ru atom of any other dendrimer. After 3 ns a group of dendrimers (02, 03, 04, 06, 07) approached, while the other dendrimers move away to rotate and find a better position in order to aggregate. After 10 ns, all the dendrimers are aggregated in a "very compact complex" that remains unchanged for the remaining 190 ns of simulation.

iii. **Self-aggregation**

With regards to the case of nine dendrimers (all different from each other, with random orientation and arranged in the center and in the vertices of a cubic box with edge l = 100 Å, in gas phase with implicit solvent), **Figure 8** shows the distances vs time between the central Ru atom of dendrimer 01 and the central Ru atom of any other dendrimer**.** After only 3 ns, a group of dendrimers (02, 03, 04, 06 and 07) approached to the dendrimer 01, while the dendrimers 05, 08 and 09 move away to rotate and find a better position in order to aggregate. After 10 ns, the position of all dendrimers is stabilized: the dendrimers are aggregated in a "very compact complex" that remains unchanged for the remaining 190 ns of simulation.

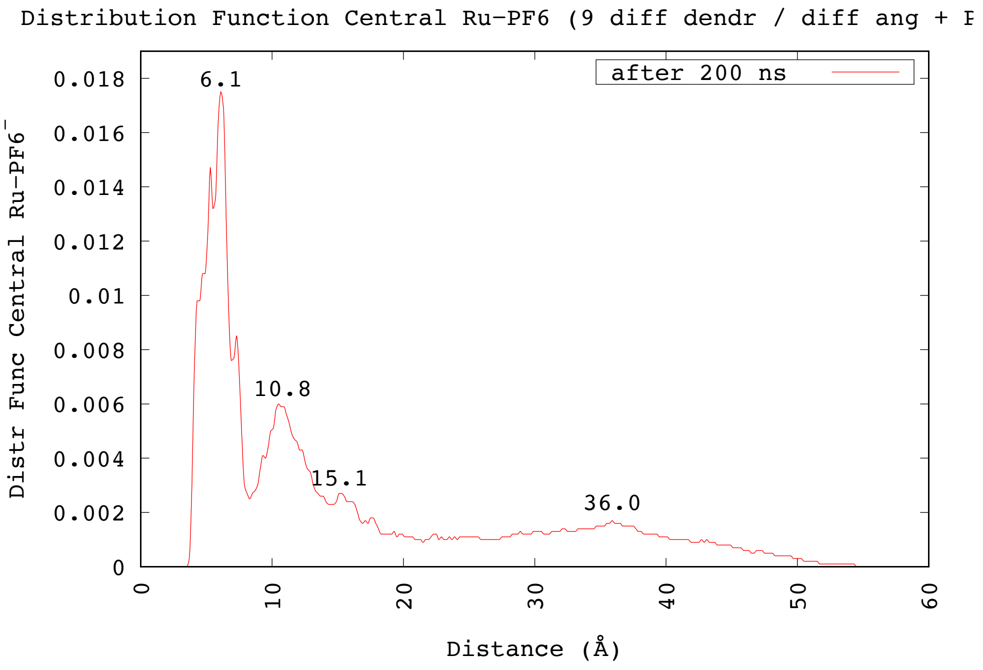

Figure 9. The distribution function of distances between Ru and PF_6^- after 200 ns.

With regard to the position of PF_6^- counterions, the distribution function of distances between ruthenium centers and PF_6^- anions shows, as in case of a single dendrimer, (see **Figure 7**) that the anions arrange near the dendrimer molecules. This can be observed both in PDDF graph (see **Figure 9**) and in snapshots of trajectory (included in the main paper).

In **Figure 9,** showing the distribution function of distances between Ru(II) centers and PF_6^- anions after 200 ns, peaks at 6.1 Å, 10.8 Å, 15.1 Å and 36 Å are evident. These peaks show that most of PF_6^- anions are arranged as close as possible to Ru atoms (i.e, 6.1 Å considering the presence of ligands). Moreover we do not find any PF_6^- anions at a distance greater than 52 Å because, when the dendrimers are aggregated, the maximum distance between their atoms is 70 Å, therefore the peak at 36 Å and the distribution function zeroed over 52 Å indicate that the anions remain as close as possible to the aggregated dendrimers.

Experimental details related to STEM experiments

Scanning Transmission Electron Microscopy (STEM) analyses were carried out with a Zeiss-Gemini 2 electron microscope, operating at 30 kV and at a working distance of 4 mm. SEM apparatus is coupled with a Quantax EDX spectrometer to carry out energy dispersive X-ray (EDX) analysis. The EDX detected pear-shaped dimension is about 0.7 mm. A drop of each suspension was deposited on a 400 mesh holey-carbon grid and left to dry at room temperature for 4 h.

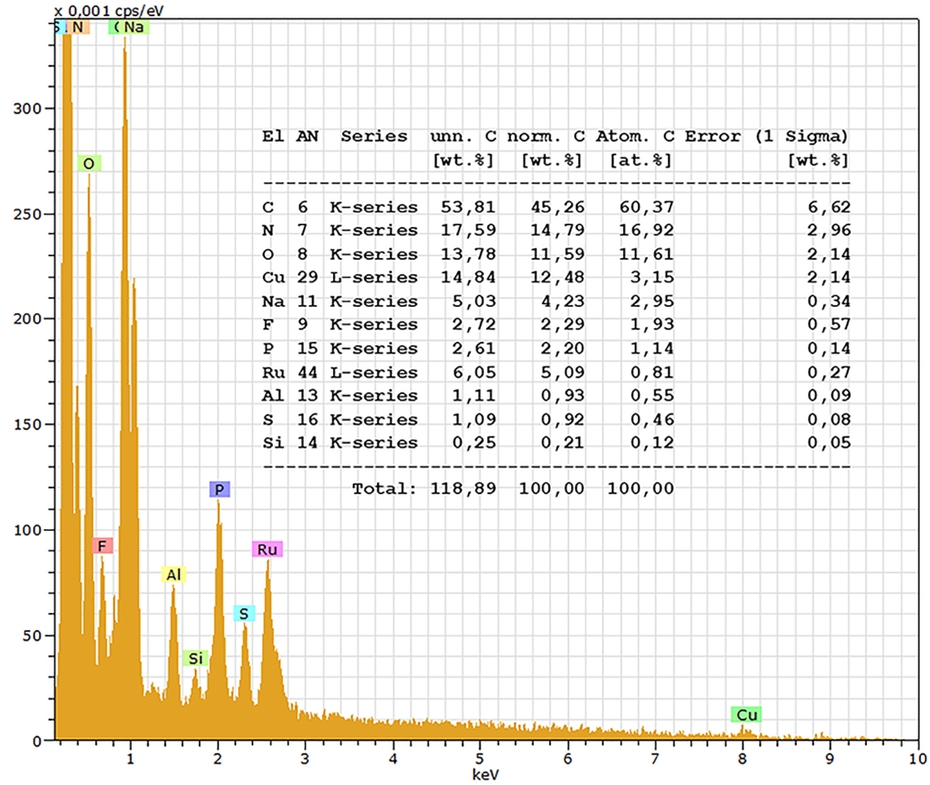

El	AN	Series	unn. C [wt.%]	norm. C [wt.%]	Atom. C [at.%]	Error (1 Sigma) [wt.%]
C	6	K-series	53,81	45,26	60,37	6,62
N	7	K-series	17,59	14,79	16,92	2,96
O	8	K-series	13,78	11,59	11,61	2,14
Cu	29	L-series	14,84	12,48	3,15	2,14
Na	11	K-series	5,03	4,23	2,95	0,34
F	9	K-series	2,72	2,29	1,93	0,57
P	15	K-series	2,61	2,20	1,14	0,14
Ru	44	L-series	6,05	5,09	0,81	0,27
Al	13	K-series	1,11	0,93	0,55	0,09
S	16	K-series	1,09	0,92	0,46	0,08
Si	14	K-series	0,25	0,21	0,12	0,05
		Total:	118,89	100,00	100,00	

Figure 10. EDX analysis obtained from diluited ($5x10^{-6}$ M) acetonitrile solution of **Ru10**.

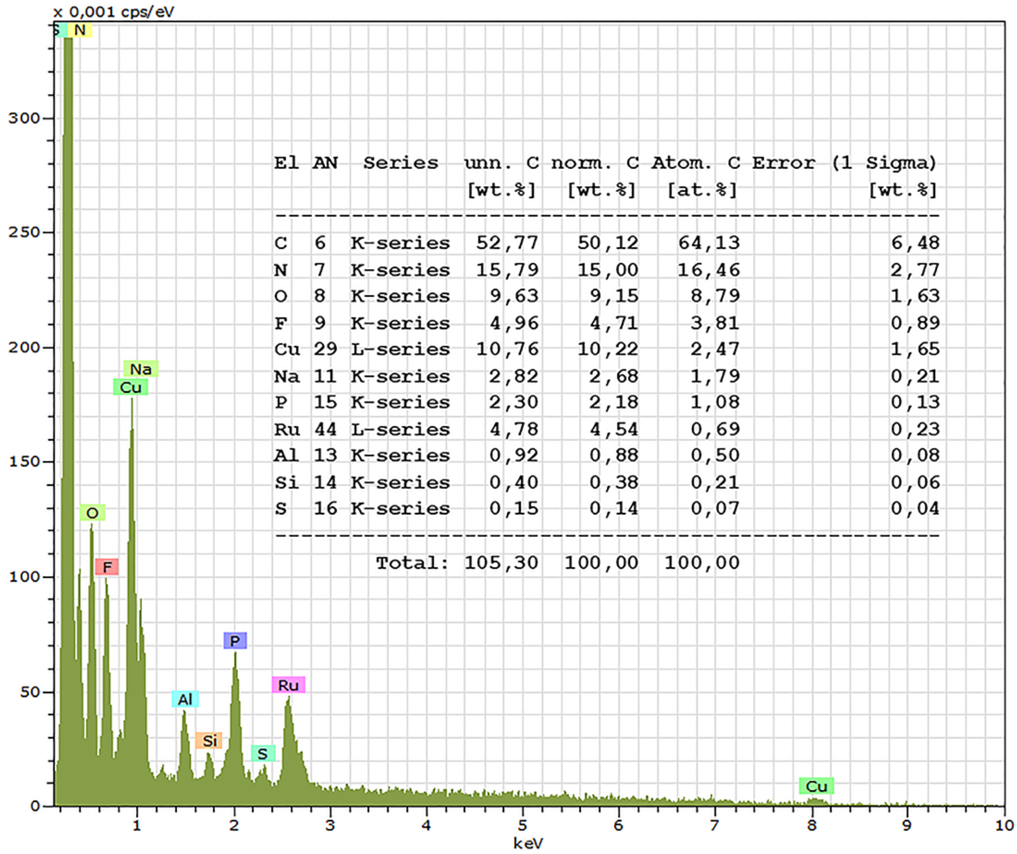

El	AN	Series	unn. C [wt.%]	norm. C [wt.%]	Atom. C [at.%]	Error (1 Sigma) [wt.%]
C	6	K-series	52,77	50,12	64,13	6,48
N	7	K-series	15,79	15,00	16,46	2,77
O	8	K-series	9,63	9,15	8,79	1,63
F	9	K-series	4,96	4,71	3,81	0,89
Cu	29	L-series	10,76	10,22	2,47	1,65
Na	11	K-series	2,82	2,68	1,79	0,21
P	15	K-series	2,30	2,18	1,08	0,13
Ru	44	L-series	4,78	4,54	0,69	0,23
Al	13	K-series	0,92	0,88	0,50	0,08
Si	14	K-series	0,40	0,38	0,21	0,06
S	16	K-series	0,15	0,14	0,07	0,04
		Total:	105,30	100,00	100,00	

Figure 11. EDX analysis obtained from concentrated (5×10^{-5} M) acetonitrile solution of **Ru10**.

References and Notes

[1] P. Brandt, T. Norrby, B. Akermark, P.-O. Norrby, *Inorg. Chem.,* **1998**, *37*, 4120.

[2] M. E. Moret, I. Tavernelli, U. Rothlisberger, *J. Phys. Chem. B*, **2009**, *113*, 7737.

[3] C. Adlhart, P. Chen, *Angew. Chem. Int. Ed.*, **2002**, *41*, 4484.

[4] W. D. Cornell, P. Cieplak, C. I. Bayly, I. R. Gould, K. M. Merz Jr., D. M. Ferguson, D. C. Spellmeyer, T. Fox, J. W. Caldwell, P. A. Kollman, *J. Am. Chem. Soc.*, **1995**, *117,* 5179.

[5] W. Humphrey, A. Dalke, K. Schulten, *J. Molec. Graphics*, **1996**, *14*, 33.

[6] F. Neese, *ORCA. An ab-initio, DFT and semiempirical SCF-MO package, Version 4.0*, Max-Planck-Institute for Chemical Energy Conversion, Mulheim-an-der-Ruhr, Germany, 2017.

[7] A. H. Goller, U. W. Grummt, *Chem. Phys. Lett.*, **2002**, *354*, 233.

[8] I. Yildirim, H. A. Stern, S. D. Kennedy, J. D. Tubbs, D. H. Turner, *J. Chem. Theory Comput.*, **2010**, *6*, 1520.

[9] *MATHLAB and Statistical Toolbox Release 2014a*. The MathWorks Inc., Natlik, Massachussets, USA.

[10] In order to transform the parameters obtained by fitting in torsion term parameters, we had to match $f(t) = \frac{a_0}{2} + \sum_{n=1}^{N} [a_n \cdot cos(nt) + b_n \cdot sin(nt)]$ with $U_{dihedral} = V_n [1 + \cos(n\phi - \phi_n)]$. We have neglected the term a_0 because it represents only a translation along the y axis. The other terms were derived as detailed:

- if $a_n \geq 0$ and $b_n \leq 0$ then $V_n = a_n$ and $\phi_n = 360 - \left|\frac{b_n \cdot 180}{\pi}\right|$
- if $a_n \leq 0$ and $b_n \geq 0$ then $V_n = |a_n|$ and $\phi_n = 180 + \left(\frac{b_n \cdot 180}{\pi}\right)$
- if $a_n \leq 0$ and $b_n \leq 0$ then $V_n = |a_n|$ and $\phi_n = 180 - \left|\frac{b_n \cdot 180}{\pi}\right|$
